# Metronomic Chemotherapy in Pediatric Oncology: From Preclinical Evidence to Clinical Studies

**DOI:** 10.3390/jcm11216254

**Published:** 2022-10-24

**Authors:** Marta Banchi, Elisabetta Fini, Stefania Crucitta, Guido Bocci

**Affiliations:** Department of Clinical and Experimental Medicine, University of Pisa, Via Roma 55, 56126 Pisa, Italy

**Keywords:** metronomic chemotherapy, pediatric tumors, preclinical studies, clinical studies, pharmacokinetic studies, biomarkers

## Abstract

Metronomic chemotherapy (MC) is the frequent, regular administration of drug doses designed to maintain a low, but active, range of concentrations of chemotherapeutic drugs, during prolonged periods of time without inducing excessive toxicities. To date, more than 400,000 children and adolescents under the age of 20 are diagnosed with cancer, per year, with 80% survival in most high-income countries, but less than 30% in low- and middle-income ones. In this review, we summarized the principal preclinical and clinical studies involving the use of MC in the most common pediatric tumors, with an overview of efficacy, toxicity, pharmacokinetic profile, and biomarkers. The best advantages of MC are low toxicity, oral administration and, thus, the feasibility of a more comfortable, home-based treatment, therefore improving the quality of life of the children themselves and of their parents and caregivers. Moreover, MC could represent a valid method to reduce the economic burden of anticancer therapy in the pediatric setting.

## 1. Introduction

**Figure 1:**
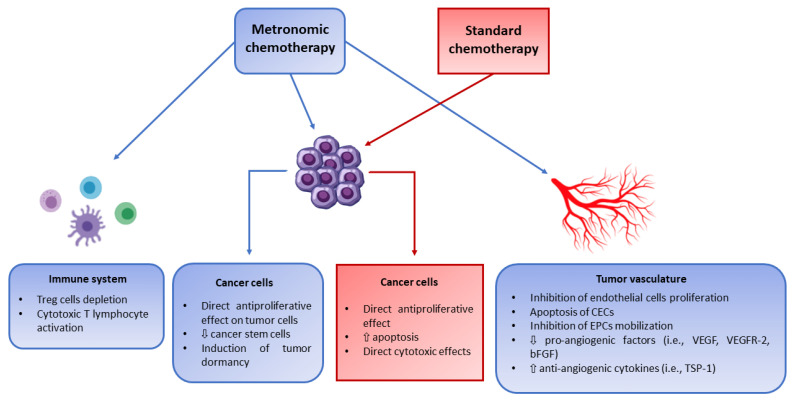
Main mechanisms of action of metronomic chemotherapy vs. standard chemotherapy. bFGF, basic fibroblast growth factor; CEC, circulating endothelial cell; EPC, endothelial progenitor cell; TSP-1, thrombospondin-1; T_reg_, regulatory T cell; VEGF, vascular endothelial growth factor.

Metronomic chemotherapy (MC) is the frequent, regular administration of drug doses designed to maintain a low, but active, range of concentrations of chemotherapeutic drugs, during prolonged periods of time without inducing excessive toxicities. MC regimens were developed to optimize the antitumor efficacy of cytotoxic agents, thus avoiding recurrent issues of standard maximum tolerated dose (MTD) chemotherapy [1]. MC acts through different mechanisms, depending on doses and schedules: the inhibition of tumor angiogenesis with a privileged activity against endothelial cells [2,3]; induction of tumor dormancy [4]; direct cytotoxic effect on cancer stem cells [5]; activation of anticancer immune response by depletion of T regulatory (Treg) cells [6] (Figure 1).

Despite major improvements in treatment and cure rates, childhood cancer incidence tends to increase with time worldwide [7]. To date, more than 400,000 children and adolescents under the age of 20 are diagnosed with cancer, per year, with 80% survival in most high-income countries, but less than 30% in low- and middle-income ones [8].

The implementation of MC in pediatric oncology, alone or in combination with other approaches such as radiotherapy, MTD chemotherapy, immunotherapy, and targeted agents, has mostly adjuvant, palliative, and maintenance purposes [9].

The main goal of administering a metronomic regimen to this specific patient population is about improving the quality of life of the children themselves and of their parents and caregivers. In fact, the best advantages of MC are low toxicity, oral administration and, thus, the feasibility of a more comfortable, home-based treatment. MC avoids the need for central venous access, contributing to a reduced risk of infection. Hematological, hepatic, or renal adverse events, after metronomic therapy, are rare and thus minimal monitoring and supportive care are necessary [10]. The lower costs, wide availability, and less need for hospitalization due to MC represent additional benefits, especially in resource-limited countries, where the majority of children with cancer live. As a result, a well-tolerated and easy-to-take metronomic treatment represents a valid strategy as maintenance or adjuvant therapy, when tumor masses are limited, or also in case of advanced disease with minimal chance of survival [10].

In this review, we summarized the major preclinical and clinical studies involving the use of MC in the most common pediatric tumors, with an overview of efficacy, toxicity, pharmacokinetic profile, and biomarkers.

## 2. Methodology

The methodology search was conducted in the PubMed database using the terms listed in Table 1, including both original articles and reviews written in the English language, published from January 2000 to September 2022. Pivotal papers published before January 2000 were also included.

## 3. Preclinical Activity of Metronomic Chemotherapy in Pediatric Tumor Models

All the described preclinical studies are summarized in Table 2.

### 3.1. Preclinical Models of Neuroblastoma

Neuroblastoma (NB) is an embryonic cancer arising from neural crest stem cells. It is the most common malignancy in infants and the most common extracranial solid tumor in children [11].

The first attempt to apply metronomic chemotherapy in these pediatric cancers was from the group guided by Robert Kerbel at the University of Toronto [12]. The xenografts of poor prognosis-related human neuroepithelioma (SK-N-MC, expressing multidrug resistance-associated protein) and neuroblastoma (SK-N-AS, highly tumorigenic) cell lines, implanted subcutaneously in CB-17 SCID mice, were continuously exposed to low doses of vinblastine (1.5 mg/m^2^ i.p. every 3 days; about 1/4 of the MTD in humans and 1/16–1/20 of the MTD in mice), DC101 (a monoclonal neutralizing antibody targeting the VEGFR-2; 800 μg/mouse, i.p. every 3 days), or their combination. Either single treatment caused a significant but short-term tumor regression, reduced tumor vascularization, and directly inhibited the angiogenic process. Interestingly, the combination induced complete and sustained tumor regression, without marked toxicity or appearance of drug resistance during more than 6 months of treatment [12]. Indeed, blocking VEGF sensitized endothelial cells to the cytotoxic effects of chemotherapy, especially when given at low doses, owing to the downregulation of various antiapoptotic proteins typically induced by VEGF itself (i.e., Bcl-2, XIAP, survivin) [13,14,15]. The microtubule-targeting agent vinblastine, given at very low doses, has been found to damage specific functions of endothelial cells, and thus angiogenesis, inducing a slight perturbance of the cytoskeleton, without evident apoptosis [16]. Rapamycin is an mTOR inhibitor with immune-suppressor activities, which directly inhibits tumor cell proliferation by arresting the cell cycle, and angiogenesis through a decrease in the production of VEGF [17]. Moreover, it inhibits the in vitro proliferation of human NB cells [18]. Marimpietri and colleagues [19] demonstrated the synergistic antiangiogenic effect of the frequent, low-dose delivery of vinblastine and rapamycin. Vinblastine (0.625–250 pM) and rapamycin (1.56–1000 pM), administered for 144 h (6 days) thrice a week, led to an in vitro antiproliferative effect on endothelial cells, starting from a concentration of 1.25 pM and 3 pM, respectively. Interaction indices showed a synergistic effect after the combination of these two agents at low doses in endothelial cells. The inhibition of proliferation was also observed in endothelial cells previously incubated with a conditioned medium from the human NB cell line HTLA-230. In the chick embryo chorioallantoic membrane (CAM) in vivo assay, each drug administered alone and, in particular, their combination inhibited the angiogenic effects induced by HTLA-230-derived conditioned media, NB cell line-derived tumor xenografts, and human NB biopsy specimens [19].

Another interesting experimental approach to neuroblastoma was performed with the camptothecin topotecan, a topoisomerase-I inhibitor. Metronomic topotecan (0.36 mg/kg, i.p. 5 times a week) was administered either alone or in combination with a humanized monoclonal anti-VEGF antibody (A4.6.1, 100 μg, i.p. twice a week) [20]. After 5 weeks of treatment, metronomic topotecan significantly suppressed the growth of human NGP-GFP neuroblastoma xenograft in athymic mice, compared with anti-VEGF treatment alone or control animals. All treated mice showed reduced tumor vascularization as opposed to the untreated ones at 6 weeks. However, tumor regrowth was observed in all treated mice at 9 weeks (3 weeks after treatment withdrawal), and it was associated with pronounced neo-angiogenesis. Interestingly, only the combination treatment was able to significantly stop the regrowth, if compared to single anti-VEGF therapy. The cooperation of low-dose chemotherapy with antiangiogenic drugs improved the efficacy and the extent of neuroblastoma tumor suppression [20].

Topotecan at low, not cytotoxic, doses has been previously shown to block the upregulation of hypoxia-inducible factor (HIF) -1α and -2α in NB cells in vitro [21]. Neuroblastoma xenografts, established by Hartwich and colleagues [22] through the injection of unmodified (CHLA-20 or NB-1691) or HIF-1α knockdown (SKNAS or NB-1691 shHIF-1α) NB cells into the retroperitoneal space of CB-17 SCID mice, were treated for 2 weeks with bevacizumab (5 mg/kg daily i.p.) or sunitinib (40 mg/kg daily by oral gavage) and low-dose topotecan (0.5 mg/kg daily i.p.) alone or in combination. Interestingly, as previously established, the introduction of low-dose topotecan to the bevacizumab and sunitinib-based antiangiogenic therapy elicited a significant reduction in tumor growth compared to either treatment alone. A similar outcome was detected in mice with HIF-1α knockdown tumors exposed to either bevacizumab or sunitinib alone, demonstrating that metronomic topotecan acted mostly via HIF-1α inhibition. Moreover, both antiangiogenic drugs caused a strong overexpression of HIF-1α-dependent growth factors, such as VEGF and GLUT3, while the addition of metronomic topotecan downregulated these two proteins [22].

*MYCN* amplification is the major genetic aberration which correlates with high-risk NB disease and poor clinical outcome [23]. This aggressive phenotype has been also associated with increased tumor neovascularization [24,25]. Taschner-Mandl and collaborators [26] found that metronomic topotecan in vitro (5 nM for 3 weeks) selectively promoted DNA damage and a tumor-inhibiting favorable senescence-associated secretory phenotype (SASP) in aggressive MYCN-amplified neuroblastoma cells (i.e., STA-NB-10 and CLB-Ma). In *MYCN*-amplified STA-NB-10 xenografts established in CD1:Foxn1^nu/nu^ mice, continuous low-dose topotecan (0.1 mg/kg/day i.p. for 6 or 15 weeks) led to tumor regression and prolonged survival, with no evident toxicity. *MYCN* mRNA and protein expression was significantly reduced both in vitro and in vivo by metronomic topotecan, which also decreased VEGF-A expression and tumor vascularization [26]. Indeed, it has been demonstrated that hypoxia halts senescence in normal and cancer cell lines [27]; thus, the downregulation of HIF-1α by topotecan [28] may also contribute to the induction of senescence.

Kumar and colleagues [29] established both subcutaneous (s.c.) NB xenografts (i.e., SK-N-BE2 and SH-SY5Y) and metastatic NB models (BE2-c and NUB-7) in NOD/SCID mice. In both in vivo modalities, the mice were treated with daily oral gavage of the antiangiogenic tyrosine kinase inhibitor (TKI) pazopanib (150 mg/kg), metronomic topotecan (1.0 mg/kg), or their combination. Low-dose topotecan plus pazopanib slowed tumor growth in subcutaneous SK-N-BE2 and SH-SY5Y tumors and limited micro-metastases in BE2-c and NUB-7 models. The in vitro proliferation assay showed no synergism between topotecan and pazopanib after 72 h of exposure; therefore, the mechanism underlying the effectiveness of the combination is probably related to the antiangiogenic activity. In fact, a significant reduction in viable CECs and CEPs and tumor microvessel density has been observed compared with control and single agents in SH-SY5Y xenografts [29].

In a following study of the same group, SK-N-BE2 xenograft–bearing mice were treated with the same aforementioned treatment schedules for 28, 56, and 80 days. Only the animals receiving metronomic topotecan plus pazopanib survived up to 80 days. Combined treatment significantly reduced microvessel density compared to control and monotherapy groups. However, enriched pericyte coverage was evident only in tumors exposed to the combination for 56 and 80 days. Additionally, the concomitant treatment upregulated the expression of hypoxia and pro-angiogenic factors (i.e., HIF-1α and VEGF), as well as that of Glut-1 and hexokinase II, which are markers of enhanced aerobic glycolysis [30].

Differently from the abovementioned combination studies, Zhang and colleagues revealed a direct synergistic antiproliferative effect in ALK^F1174L^-mutated SH-SY5Y cells when exposed for 6 days to increasing concentration of crizotinib, a double inhibitor of c-MET and ALK receptor tyrosine kinases, in combination with metronomic topotecan [31]. Interestingly, the same authors did not observe any synergism in the *MYCN*-amplified SK-N-BE2, KELLY, and LAN-5 cells, suggesting that *MYCN* is potentially involved in the emergence of resistance to ALK inhibitors. In vivo, crizotinib (50 mg/kg) and metronomic topotecan (1 mg/kg) were administered daily for 9 days by oral gavage, alone or in combination, in ALK^F1174L^-mutated SH-SY5Y and KELLY mouse xenografts. The combined treatment significantly delayed tumor growth derived from KELLY cells, whereas it induced the complete regression of SH-SY5Y tumors [31].

Although metronomic topotecan and vinblastine are the chemotherapeutic drugs that have been most frequently used in preclinical models of neuroblastoma, other metronomic therapeutic approaches have been tested with metronomic cyclophosphamide (mCTX) at the dose of 40 mg/kg/day p.o. in SH-SY5Y (chemotherapy-sensitive, non-*MYCN*-amplified) and SK-N-BE2 (chemotherapy-resistant, *MYCN*-amplified) tumor xenografts. Morscher and colleagues [32] observed a greater inhibition of tumor expansion in *MYCN*-amplified mCTX-treated xenografts, as well as a considerable diminution of blood vessel density and intratumoral bleeding. Moreover, they detected a decreased Bcl-2 expression and elevated caspase-3 cleavage. In contrast, non-*MYCN*-amplified tumors showed upregulation of Bcl-2 and developed resistance [32]. Intriguingly, combining mCTX with a calorie-restricted ketogenic diet [33] significantly enhanced the antitumor effects of this therapeutic approach, resulting in tumor regression and the complete growth arrest of both NB xenografts [32]. However, calorie restriction would not be recommended in most young oncologic patients. Optimization of KD with 25% of 8-carbon medium-chain triglycerides proved to increase the efficacy of mCTX therapy (oral dose of 40 and 13 mg/kg/day given to SH-SY5Y- and SK-N-BE2-bearing mice, respectively), similarly to that achieved with calorie-restricted KD. This combination induced a significant suppression of tumor growth and prolonged survival, especially in SH-SY5Y xenografts. Furthermore, it caused an obvious inhibition of angiogenesis in vivo and activation of AMPK in NB cells. These data highlight that the metabolic stress, induced by dietary manipulation, selectively sensitized the NB cells to low-dose chemotherapy [33].

### 3.2. Pediatric Brain Tumor Models

Pediatric cancers of the central nervous system (CNS) are the most common solid tumors in children and the second most recurrent childhood malignancies. Medulloblastoma is an embryonal tumor of the posterior fossa, and it accounts for nearly 20% of all pediatric brain tumors, making it the most common malignant brain tumor in children. Almost 20% of all childhood gliomas are high-grade gliomas, and include anaplastic astrocytoma (AA), diffuse intrinsic pontine glioma (DIPG), and glioblastoma multiforme (GBM). Ependymoma, representing around 8% to 10% of all childhood CNS tumors, is the third most frequent brain tumor in children. Unlike adult patients with GBM, where the standard of care is represented by the combination of temozolomide and radiotherapy [34], there is currently no similar recommendation for chemotherapy in the management of pediatric high-grade glioma [35].

PEX is a fragment of human metalloproteases-2 that has significant antimitotic, anti-invasive, and antiangiogenic activities against human glioblastoma cells in vitro and glioblastoma models in vivo [36]. The research of Bello and collaborators [37] suggested that combining low and semicontinuous chemotherapeutic drugs for more than 120 days, such as carboplatin and etoposide (bolus at day 1 with 6 mg/kg carboplatin i.p. and 4 mg/kg etoposide i.p., followed by 2 mg/kg carboplatin + 2 mg/kg etoposide), with PEX (2 mg/kg of PEX i.p. for 2 days, every 3 days) was more effective than chemotherapy alone at a low dose, in the treatment of nude mice bearing intracranial human U87 glioblastoma xenografts. This regimen was accountable for better survival, substantial reduction in tumor volume, vascularization and proliferation index, increased apoptosis, and no complications [37].

Folkins and colleagues [5] focused their investigations on the effect of antiangiogenic therapy on glioma tumor stem-like cells (TSLCs). Actually, there is evidence that brain TSLCs are preserved, such as a neural stem cell, by a vascular niche, and consequently have the possibility to stimulate tumor growth. The researchers treated athymic nude mice bearing s.c. rat C6 glioma xenografts, with either anti-VEGFR-2 antibody DC101 (800 μg/mouse i.p. every 3 days), low dose metronomic (LDM) cyclophosphamide (20 mg/kg/day p.o. via the drinking water), MTD cyclophosphamide (100 mg/kg i.p. on days 1, 3, and 5 of a 21-day cycle), or combinatorial regimes. Antiangiogenic or cytotoxic therapy alone was not sufficient to reduce the amount of glioma TSLCs. Interestingly, the combination of LDM cyclophosphamide with the potent inhibition of angiogenesis by DC101 was the best therapeutic option for the significant and selective elimination of TSLCs from glioma. Therefore, antiangiogenic therapy acquires a potential new role in chemo-sensitizing TSLCs, in order to improve the effectiveness of chemotherapy [5].

Topotecan has shown to inhibit HIF-1α protein amassing in human cancer cell lines [28] independently of replication-mediated DNA damage, suggesting the existence of an alternative mechanism of action to the cytotoxic one. In their study, Rapisarda and colleagues showed that low concentrations of topotecan delivered on a daily, but not intermittent, schedule (1 mg/kg, 10 total doses) caused a sustained inhibition of tumor progression in human U251-HRE glioblastoma xenografts. Topotecan exerted its antitumor activity through the prominent diminution of the HIF-1α protein amount, angiogenesis, and expression of genes targeted by HIF-1 in the tumor mass, compared to untreated controls [38].

In a subsequent work, the same investigators found that combining a very low dose of topotecan (0.5 mg/kg once a day, for 10 days) with bevacizumab significantly suppressed glioblastoma growth, as opposed to either agent alone. Whereas bevacizumab alone induced the expression of HIF-1-dependent genes, the addition of topotecan clearly abrogated HIF-1 transcriptional activity in the tumor microenvironment, and significantly inhibited proliferation and induced apoptosis of tumor cells [39].

The alkylating agent temozolomide is one of the most successful chemotherapeutic drugs against glioblastoma, but its antitumor activity is often limited by the onset of resistance. Kim and collaborators showed that C6/LacZ rat glioma cells were more resistant to LDM temozolomide (1–100 μM daily for 144 h), with a ~10-fold greater IC_50_ value than U-87MG human glioblastoma cells. In an orthotopic SD rat model of C6/LacZ glioma, LDM temozolomide (2 mg/kg p.o. daily for 16 days) significantly hampered tumor growth and angiogenesis and promoted apoptosis, compared to the conventional schedule (7 mg/kg for 5 days). In an orthotopic nude mouse xenograft of U-87MG glioblastoma, despite no significant changes in tumor volume between conventional and metronomic regimens, the periodic administration of temozolomide even at a very low dose (0.25 mg/kg daily for 25 days) markedly reduced the microvessel density. In summary, LDM temozolomide demonstrated a striking antiangiogenic effect, either in resistant or sensitive glioma models, suggesting the possibility to overcome resistance in standard temozolomide chemotherapy [40].

Banissi and colleagues studied the impact of LDM versus standard temozolomide regimens on the regulatory T cell (Treg) fraction in Fischer rats bearing an s.c. temozolomide-resistant RG2 glioma tumor. Temozolomide significantly decreased the Treg/CD4+ T cell proportion when given at very low doses (0.5 or 2 mg/kg/die, 5 days per week for 21 days), but not at high doses (30 mg/kg/die for 5 days, or 10 mg/kg/die, 5 days per week for 21 days). Such Treg exhaustion was marked among the splenic lymphocyte population, and it was almost significant among the tumor-infiltrating lymphocytes. However, Treg depletion alone, detected in LDM temozolomide-treated rats, was not sufficient to significantly inhibit tumor progression as well, in comparison with control animals. In conclusion, LDM, but not conventional, schedules of temozolomide showed selective cytotoxicity to the circulating Tregs population [6].

Morphine is the most used drug for pain management in oncological patients. The preclinical studies investigating the effect of opioid receptor agonists on the proliferation, migration, and invasion of cancer cells, as well as their immunosuppressive and proangiogenic activities, show conflicting results. It is presumed that the activation of μ-opioid receptor (MOP) and Toll-like receptor 4 receptors may contribute to tumor growth and progression, while the activation of κ-opioid receptor may promote anticancer and antiangiogenic effects [41]. It has been highlighted that methadone, an agonist of MOP, increased the sensitivity of wild-type leukemic cells, but not MOP-depleted cells, to L-asparaginase treatment. Therefore, MOP was necessary for the synergistic action of L-asparaginase and methadone, and MOP loss promoted leukemic cell survival likely through the downregulation of the MOP-mediated apoptotic pathway [42]. For the first time, Iorio and colleagues demonstrated that morphine acts as an inhibitor of P-glycoprotein, an ATP-binding cassette transporter overexpressed in the endothelial cells of the blood brain barrier and involved in resistance to many chemotherapeutic drugs (i.e., temozolomide). Therefore, morphine might increase the effects of chemotherapy on brain tissue. The authors showed that LDM temozolomide (1.77 mg/kg/day) was effective, from the beginning of the treatment, in hampering the growth of an orthotopic human U87MG-luc2 glioblastoma xenograft, implanted intracranially in Foxn1 nude mice. Noteworthy, combining temozolomide (1.77 and 0.9 mg/kg/day, for a total of 5 weeks) with weekly morphine enhanced the antitumor efficacy and the long-term response to the metronomic regimens in this glioblastoma model [43].

Chen and colleagues described a peculiar metronomic schedule, named MEDIC (medium-dose intermittent chemotherapy), consisting of an immunogenic chemotherapeutic agent (i.e., cyclophosphamide), given at a concentration between a daily low dose (i.e., 20–25 mg/kg) and an MTD dose (i.e., 150–170 mg/kg × 2 or 3 consecutive days, every 21 days), with a medium-term drug-free break and low systemic toxicity. The MEDIC regimen, in addition to the direct cytotoxic effect on tumor cells, can trigger a prolonged antitumor immune response [44].

In murine s.c. models of rat 9L gliosarcoma, mouse GL261 glioma, and human U251 glioblastoma, cyclophosphamide (140 mg/kg i.p.), given on an intermittent metronomic schedule (every 6 days), activated robust and persistent immune responses, as well as induced a striking and extended tumor regression, even with the lack of an evident antiangiogenic effect. Conversely, the MTD regimen caused temporary immune responses, concurrently with appreciable tumor re-growth [44,45,46]. Interestingly, the MEDIC protocol was also more effective than the corresponding daily low-dose metronomic regimen [44]. In addition, it stimulated a strong CD8+ T cell response leading to tumor disappearance and the gain of immune memory in an immunocompetent, syngeneic s.c. GL261 glioma [47]. Suppression of immune activity occurred if the drug-free interval lasted more than 6 days, as indicated by a significant increase in Foxp3+ Treg cells within the tumor and decreased expression of the cytotoxic immune mediator perforin [47].

Ferrer-Font and collaborators [48] aimed to assess if the every 6-day metronomic regimens of cyclophosphamide (140 mg/kg) or temozolomide (140, 200, and 240 mg/kg) were also effective in an immunocompetent orthotopic GL261 mouse model. They found a longer survival rate in mice treated with both regimes than in control animals. The best results were obtained with 140 mg/kg temozolomide, which resulted in the best dosing in the reduction in tumor volume and improved survival, and in comparison with a previously tested non-metronomic schedule of this drug (60 mg/kg for three cycles on days 11–15, 19–20, and 24–25) [49]. However, in contrast with the results described by Wu and Waxman, no tumor eradication was achieved in this model, highlighting the existence of relevant differences in the tumor environment between ectopic and orthotopic glioblastoma xenografts, which may influence the response to metronomic chemotherapy [47].

It has been demonstrated that there is an immunomodulatory—dosing dependent—effect of temozolomide, which could impact the response to immunotherapy. In GL261 and KR158 murine intracranial glioma models, the standard (50 mg/kg × 5 days), but not metronomic (25 mg/kg × 10 days), regimen of temozolomide led to overexpression of exhaustion markers (i.e., TIM-3 and LAG-3) on peripheral and tumor-infiltrating T cells and to an increase in immunosuppressive cells. Interestingly, the addition of metronomic temozolomide to PD-1 immune checkpoint inhibition preserved the survival benefit gained with anti-PD-1 therapy alone in the GL261 model, while it was abolished by standard temozolomide. Therefore, these findings suggest metronomic dosing of temozolomide as a preferential strategy in chemoimmunotherapy regimens for the management of glioblastoma [50].

### 3.3. Soft Tissue and Bone Sarcoma Models

Soft tissue sarcomas represent 7.4% of cancer cases in children younger than 20 years of age. Rhabdomyosarcoma is the most common (50%) soft tissue sarcoma among children less than 14 years old [51]. Ewing sarcoma and osteosarcoma are the two most commonly diagnosed bone cancers in pediatric patients [52].

Continuous low-dose doxorubicin (1.2 mg/kg, twice weekly for 4 weeks) was found to modestly inhibit the tumor growth of human rhabdomyosarcoma RD s.c. xenografts, well-established in SCID mice, corresponding to 46.5% of that achieved with the standard treatment (6 mg/kg once every 2 weeks). Moreover, the development of human leiomyosarcoma SKLMS-1 s.c. tumors was not halted by metronomic doxorubicin. However, the combination of low-dose doxorubicin and DC101 at a lower concentration (400 μg/dose every 3 days for a total of seven times) markedly reduced the volume of both SKLMS-1 and RD tumors, compared to either agent alone. Microvessel counts were significantly lower in combination-treated mice, compared to either agent alone, and no further toxicity was detected as opposed to low-dose doxorubicin alone. Moreover, DC101 plus doxorubicin exerted a direct additive inhibitory effect on endothelial cell functions (i.e., migration, proliferation, tube-like formation) in vitro and enhanced apoptosis of endothelial cells, through caspase-3 activation. These results support antiangiogenic activity as one of the main mechanisms for the antitumor effect of combined low-dose treatment with DC101 and doxorubicin [53].

Osteosarcoma UMR 106-bearing SD rats were treated, for a period of 8 weeks, with: (i) a conventional schedule including a high dosage of methotrexate (1.35 g/kg i.v., 6 h infusion, once weekly, at week 0, 1, 5 and 6), adriamycin and cisplatin (10 mg/kg and 20 mg/kg, respectively; once weekly, at week 2 and 7); (ii) a metronomic schedule of low-dose methotrexate (1.2 mg/kg i.v., bolus, twice a week); and (iii) a combination schedule of metronomic and conventional chemotherapy, with low-dose methotrexate administered at week 3, 4, and 8 of the conventional scheduling. After the first 6 weeks of treatment, in which the rate of tumor inhibition was similar for all three regimens, only the combination showed a protracted inhibitory effect on tumor growth. Anyway, the metronomic protocol had a better effect compared to the conventional one. Furthermore, both metronomic and combination schedules showed a much lower VEGF-A expression than the conventional group, which may support a greater antiangiogenic effect [54].

### 3.4. Pediatric Retinoblastoma Models

The standard chemotherapy for retinoblastoma, the most common intraocular cancer of childhood [55], includes intravenous or local intravitreal injections of melphalan, carboplatin, and topotecan, which unfortunately induce retinal toxicity [56].

Both commercial (Y79 and WERI-RB1) and patient-derived retinoblastoma cell lines (HSJD-RBT-7 and HSJD-RBT-8) together with human vascular endothelial cells (HUVEC and EPC) were exposed to increasing concentrations of melphalan or topotecan in a conventional (0.001–1000 μM or 0.001–10.000 nM, respectively, single 72 h exposure) or metronomic (0.0001–100 μM or 0.0001–1000 nM, respectively, 7-day continuous exposure) treatment protocol. The continuous administration of melphalan and topotecan increased the sensitivity of retinoblastoma and endothelial cell lines with significant lower IC_50_ values (i.e., 18.4- and 12.3-fold for topotecan and 8.3- and 13.5-fold for melphalan in Y79 and WERI-RB1, respectively) compared to conventional treatment. The cytotoxic effect of metronomic chemotherapy was more evident in commercial cell lines compared to the patient-derived HSJD-RBT-8 cells. Furthermore, the heavily pretreated HSJD-RBT-8 cells had enhanced chemosensitivity to the metronomic schedule (3 to 4-fold decreased IC_50_) compared to conventional dosing, while no change was evident for naïve HSJD-RBT-7 cells, suggesting a possible mechanism to overcome resistance. Both treatment regimens led to cell death through apoptosis and/or necrosis in all cell lines. Moreover, metronomic topotecan or melphalan significantly inhibited in vitro tube formation in HUVEC and EPC compared to control cells. In athymic nude mice harboring a Y79 retinoblastoma xenograft, low-dose topotecan (0.6 mg/kg i.p., 5 days a week for 2 weeks) determined significantly lower tumor volumes compared to the MTD protocol (3 mg/kg i.p., once a week for 2 weeks) and the control group after 14 days of treatment, with a similar toxicity profile and no weight loss. These results highlighted the dual cytotoxic/antiangiogenic, schedule-dependent effect of chemotherapy in retinoblastoma preclinical models. [57].

### 3.5. Acute Lymphoblastic Leukemia Models

Acute lymphoblastic leukemia (ALL) is the most common pediatric malignancy, representing 75%–80% of acute leukemias among children, with a median age at diagnosis of 15 years [58]. Glucocorticoid (GC) resistance represents a crucial challenge in treating ALL; therefore, there is an urgent need for new therapeutic strategies to enhance chemosensitivity, which may also allow for reduction in the intensity and toxicity of standard chemotherapy [59].

It has been demonstrated that a low, subtoxic concentration of arsenic trioxide (0.25 μM) significantly increased in vitro dexamethasone sensitivity of 3 different GC-resistant ALL cell lines (CEM-C1-15, Jurkat, and MOLT-4), as well as T-ALL and precursor B-ALL cells from pediatric patients with poor response to prednisone. The effect of this combination was explained in part by a reduction in Akt phosphorylation and following alteration of downstream Akt targets such as an increase in Bad, a proapoptotic Bcl-2 family member, and a decrease in the X-linked inhibitor of apoptosis protein (XIAP). These results indicate that a combination of low-dose arsenic trioxide with glucocorticoids may be a valuable approach to reverse GC resistance and improve prognosis in ALL pediatric patients [60].

Next, 2-deoxy-D-glucose (2-DG) is a glucose analogue that inhibits the proliferation of cancer cells through the inhibition of glycolysis and N-linked glycosylation [61]. Recently, Gu and co-workers showed that a low, nontoxic dose of 2-DG (1 mM for 48 h) was able to induce apoptosis and cell-cycle arrest and to overcome GC resistance in ALL cells under normoxia. In fact, low-dose 2-DG combined with dexamethasone (1 μM) recovered the sensitivity of GC and produced a strong synergistic cytotoxic effect in GC-resistant Molt-4 (T lineage) and Raji (B lineage) cells. Interestingly, these effects were achieved mainly by blocking N-Linked glycosylation and inducing endoplasmic reticulum stress [62].

L-asparaginase (L-ASNase) is a critical anticancer agent used in the treatment of ALL and some types of non-Hodgkin’s lymphoma, including natural killer (NK)-cell lymphoma [63]. Many leukemia or lymphoma cells rely on the supply of the amino acid asparagine (Asn) from plasma, due to a deficiency of l-asparagine synthetase [64]. L-ASNase hydrolyzes L-Asn to L-aspartic acid, therefore, lowering the level of plasma Asn, which leads leukemic cells to apoptosis [65]. L-ASNase also shows some glutaminase (GLS) activity, that converts the circulating glutamine (Gln) to glutamate and ammonia, thus reducing Gln intracellular uptake. Interestingly, low doses (0.01 U/mL for 24 h) of L-ASNase induced Asn depletion and effectively killed Asn-dependent NK-YS cells, whereas clinically achievable intermediate doses (1 U/mL for 24 h) caused Gln depletion and robust apoptosis in Gln-dependent ALL Jurkat and mantle cell lymphoma Jeko cell lines. In addition, the high expression of glutaminase GLS1 was found to be related to increased sensitivity to L-ASNase in pediatric B lineage ALL [66].

### 3.6. Metronomic Combined Schedules in Multiple Pediatric Tumor Models

Zhang and collaborators revealed that low-dose topotecan (20 nmol/L) significantly enhanced the cytotoxic effect of the hypoxia-activated prodrug (HAP) evofosfamide on a wide range of neuroblastoma and rhabdomyosarcoma cell lines, in in vitro 72 h exposure experiments. Aggressive neuroblastoma (CHLA-20 and SK-N-BE(2)), rhabdomyosarcoma (RH4 and RD) s.c. xenografts, and the neuroblastoma SK-N-BE(2) metastatic model were subsequently treated with evofosfamide, (50 mg/kg daily i.p., 5 days/week), LDM topotecan (1 mg/kg daily by oral gavage, 5 days/week), and their combination. In every neuroblastoma and rhabdomyosarcoma xenograft, the combined treatment resulted in a better antitumor effect than both monotherapies and induced complete tumor regression after 2 weeks of treatment. The combination also improved survival in the SK-N-BE(2) metastatic model (median survival of 46 days), compared to either agent alone. There was no serious toxicity related to therapy in any of the experimental models. Interestingly, in RH4 xenografts, evofosfamide induced apoptosis of tumor cells localized mostly in hypoxic regions, while topotecan targeted tumor cells mainly within normoxic areas. Thus, administering evofosfamide with LDM topotecan allowed it to kill tumor cells in both normoxic and hypoxic regions, explaining the higher efficacy of the combination regimen in all tumor models [67].

Talazoparib is a potent, selective PARP1/2 inhibitor and shows strong PARP trapping activity, which means it firmly traps PARP1 to the sites of DNA single-strand breaks [68,69]. Methylating agents (i.e., temozolomide) are extremely useful at prompting single-strand breaks that could become substrates for PARP trapping [70]. Thus, Smith and collaborators evaluated the in vitro 96 h exposure of temozolomide (0.3–1000 μmol/L) in the presence of talazoparib (10 nmol/L), which resulted in a marked potentiation of temozolomide toxicity in Ewing sarcoma (50-fold) and ALL (30-fold) cell lines. In vivo, several pediatric s.c. xenograft models (including Ewing sarcoma, Wilms tumor, rhabdomyosarcoma, osteosarcoma, neuroblastoma, brain tumors) were treated with temozolomide (30 mg/kg/day for 5 days) and talazoparib (0.25 mg/kg twice daily for 5 days) alone or in two different combinations: high-dose temozolomide (30 mg/kg/day for 5 days) + talazoparib (0.1 mg/kg twice daily for 5 days), or low-dose temozolomide (12 mg/kg/day for 5 days) + talazoparib (0.25 mg/kg twice daily for 5 days). Toxicity was comparable for both combinations. Unlike single treatments, both combinations exhibited significant antitumor effects and induced total tumor regression in 5 out 10 Ewing xenografts tested (TC-71, CHLA-258, SKNEP-1, ES-4, and ES-7), within 6 weeks of treatment. Combined treatments were also successful against pediatric preclinical models with low MGMT expression (i.e., GBM2 glioblastoma and Rh28 rhabdomyosarcoma), responsive to temozolomide, and those with a defective homologous recombination, responsive to talazoparib (i.e., KT-10 Wilms tumor). In conclusion, administering an efficacious PARP trapping concentration of talazoparib plus low-dose temozolomide is a promising strategy for translation to the pediatric clinical setting [71].

Pawlik and co-workers [72] found IC_50_ values (~2 nM) for medulloblastoma Daoy cells exposed to topotecan for a total of 6 days, in three different ways of delivering: continuous, for 8 h daily, or for 8 h every other day. In contrast, topotecan IC_50_ increased with extending the drug-free time in Rh30 rhabdomyosarcoma cells (3.9, 8.7, and 12.4 nM, respectively). In vivo experiments confirmed in vitro results. Rh30 xenografts regressed completely when treated with topotecan 0.6 mg/kg i.v. daily (dx5), whereas no growth delay was observed with 1.0 mg/kg every other day (for 2 weeks, repeated every 21 days for 3 cycles). Increased doses of topotecan 2 mg/kg daily and 3.3 mg/kg every other day, which produced comparable antitumor activity, were needed to obtain complete tumor regression of the less sensitive in vivo Daoy models. These results clearly show that the antitumor effect of topotecan was extremely schedule-dependent in Rh30, but not in Daoy cells and xenograft models.

**Table 2:** Preclinical studies of metronomic chemotherapy in pediatric tumor models.

Metronomic Regimen	Preclinical Tumor Model	Results	Reference
Neuroblastoma			
Vinblastine (1.5 mg/m^2^ i.p. every 3 days), anti-VEGFR-2 antibody DC101 (800 μg/mouse, i.p. every 3 days), or combination, for >6 months	Human SK-N-MC neuroepithelioma and SK-N-AS neuroblastoma cell lines implanted s.c. in CB-17 SCID mice, aged 4 to 6 weeks	Combined treatment induced complete and sustained tumor regression, without marked toxicity or appearance of drug resistance.	Klement et al. [12]
Topotecan (0.36 mg/kg, i.p. 5x/week), anti-VEGF antibody A4.6.1 (100 μg, i.p. twice a week), or combination for a total of 5 weeks	Human NGP-GFP neuroblastoma xenograft in female NCR athymic mice, aged 4 to 6 weeks	LDM topotecan either with or without anti-VEGF antibody significantly suppresses NB xenograft growth. All treated mice showed reduced tumor vascularization. Only combined treatment significantly stopped the regrowth. LDM topotecan increased apoptosis of neuroblastoma cells.	Kim et al. [20]
Vinblastine (0.625–250 pM) and rapamycin (1.56–1000 pM), administered for 144 h, thrice a week	In vitro ECs (HUVEC and EA.hy926) and ECs preincubated with CM from the human NB cell line HTLA-230. In vivo CAM assay with HTLA-230-derived CM, HTLA-230-derived tumor xenografts, and human NB biopsy specimens	Significant antiproliferative effect in ECs preincubated with HTLA-230 CM after combination at low doses. Combination of 50 pM vinblastine and 0.5 nM rapamycin was synergistic in arresting the cell cycle and increased apoptosis of ECs. The combination markedly inhibited the angiogenic effects induced in vivo in the CAM assay.	Marimpietri et al. [19]
Topotecan (0.5 mg/kg daily i.p.), bevacizumab (5 mg/kg daily i.p.) or sunitinib (40 mg/kg daily p.o.), or combination, for 2 weeks	Unmodified CHLA-20 and NB-1691, and shHIF-1α-modified NB-1691 and SKNAS neuroblastoma cell lines injected into the retroperitoneal space of CB-17 SCID mice	Combined LDM treatment significantly reduced tumor growth and downregulated the expression of VEGF and GLUT3.	Hartwich et al. [22]
In vitro: topotecan (5 nM, 2×/week for 3 weeks) In vivo: topotecan (0.1 mg/kg/day i.p. for 6 or 15 weeks)	In vitro WT or *MYCN*-amplified neuroblastoma cell lines (i.e., STA-NB-10 and CLB-Ma). In vivo STA-NB-10 xenografts established in female CD1: *Foxn1^nu/nu^* mice, 6 to 10 weeks old	Induction of DNA-damage and a tumor-inhibiting favorable SASP selectively in *MYCN*-amplified cells in vitro. Tumor regression and prolonged survival, with no evident toxicity in vivo. Significant reduction of MYCN mRNA and protein expression both in vitro and in vivo Decreased VEGF-A expression and tumor vascularization in vivo.	Taschner-Mandl et al. [26]
Cyclophosphamide (mCTX, 40 mg/kg/day, p.o. through the drinking water), calorie restricted KD, optimized KD, or combination	Non-MYCN-amplified SH-SY5Y and MYCN-amplified SK-N-BE(2) NB cell lines injected s.c. in female CD1 nude mice, aged 5 to 6 weeks	mCTX induced greater tumor inhibition in MYCN-amplified xenografts, diminution of blood vessel density, and intratumoral bleeding, decreased Bcl-2 expression, and increased caspase-3 cleavage. Combining mCTX with calorie restricted KD resulted in tumor regression and complete growth arrest of both NB xenografts. Combining mCTX with optimized KD resulted in significant tumor growth suppression and prolonged survival, especially in SH-SY5Y xenografts, and inhibition of angiogenesis.	Morscher et al. [32] Aminzadeh-Gohari et al. [33]
Topotecan (1.0 mg/kg/day p.o.), pazopanib (150 mg/kg/day p.o.), or combination	SK-N-BE(2) and SH-SY5Y s.c. (into inguinal fat pad) NB xenografts, BE(2)-c and NUB-72 metastatic (into lateral tail vein) NB xenografts in NOD/SCID mice	Combined treatment slowed tumor growth in SK-N-BE(2) and SH-SY5Y models, and limited micrometastasis in BE(2)-c and NUB-7 models. Significant reduction in viable CECs and CEPs and tumor microvessel density in SH-SY5Y xenografts.	Kumar et al. [29]
Topotecan (1.0 mg/kg/day p.o.), pazopanib (150 mg/kg/day p.o.), or combination for 28, 56, and 80 days	SK-N-BE(2) s.c. xenograft–bearing NOD/SCID mice, 4 to 8 weeks old	Only the animals receiving the combination survived up to 80 days. All three durations of combined treatment significantly reduced microvessel density. Higher proliferative and mitotic indices after 28 days of combined treatment.	Kumar et al. [30]
In vitro: crizotinib, LDM topotecan, or combination (ratio 20:1, 0,01–10 μM) continuously for 6 days In vivo: topotecan (1 mg/kg/day p.o.), crizotinib (50 mg/kg/day p.o.) for 9 days	In vitro ALK^F1174L^-mutated SH-SY5Y cell line and MYCN-amplified SK-N-BE(2), KELLY, and LAN-5 cell lines. In vivo ALK^F1174L^-mutated SH-SY5Y and KELLY xenografts in female NOD/SCID mice, 4 to 6 weeks old.	Combined treatment resulted in a synergistic antiproliferative effect only in SH-SY5Y cells in vitro and a significantly delayed tumor growth of KELLY xenografts, and complete regression of SH-SY5Y tumors in vivo.	Zhang et al. [31]
Brain tumor			
Bolus at day 1 with 6 mg/kg carboplatin i.p. and 4 mg/kg etoposide i.p., followed by 2 mg/kg carboplatin + 2 mg/kg etoposide and 2 mg/kg of PEX i.p. for 2 days, every 3 days. Treatment duration ≥ 120 days	Intracranial human U87 glioblastoma xenografts in 5-week-old Swiss male nude mice	This combination was more effective than chemotherapy alone and induced better survival, substantial reduction in tumor volume, vascularization and proliferation index, increased apoptosis, and no complications.	Bello et al. [37]
DC101 (800 μg/mouse i.p. every 3 days), LDM cyclophosphamide (20 mg/kg/day p.o. via the drinking water), MTD cyclophosphamide (100 mg/kg i.p. on days 1, 3, and 5 of a 21-day cycle), and their combinations	Athymic nude mice bearing s.c. rat C6 glioma xenografts	The combination of LDM cyclophosphamide with potent inhibition of angiogenesis by DC101 caused a significant and selective elimination of TSLCs from the glioma.	Folkins et al. [5]
Low-dose topotecan (1 mg/kg daily, 10 total doses)	Female athymic nude (NCr/nu) mice bearing human U251-HRE glioblastoma xenografts	Sustained inhibition of xenograft tumor progression, with prominent diminution of HIF-1α protein amount, angiogenesis, and expression of genes targeted by HIF-1 in tumor mass	Rapisarda et al. [38]
Very low-dose topotecan (0.5 mg/kg daily, for 10 days) + bevacizumab	Female athymic nude (NCr/nu) mice bearing human U251-HRE glioblastoma xenografts	The combination significantly suppressed glioblastoma growth. Addition of topotecan clearly abrogated HIF-1 transcriptional activity in the tumor microenvironment, significantly inhibited proliferation, and induced apoptosis.	Rapisarda et al. [39]
In vitro: LDM TMZ (1–100 μM daily for 144 h) In vivo rat model: conventional TMZ (7 mg/kg p.o. for 5 days) vs. LDM TMZ (1 or 2 mg/kg p.o., every day for 16 days) In vivo mouse model: conventional TMZ (2.5 or 1.25 mg/kg p.o., for 5 days) vs. LDM TMZ (0.5 and 0.25 mg/kg p.o., daily for 25 days)	In vitro C6/LacZ rat glioma cells and U-87MG human glioblastoma cells. In vivo male Sprague-Dawley (SD) rats (200–250 g) bearing intracranial C6/LacZ glioma and male Balb/c-nu mice (6 weeks) bearing intracranial U-87MG glioblastoma	In vitro C6/LacZ rat glioma cells were more resistant to LDM TMZ than U-87MG human glioblastoma cells. In the orthotopic C6/LacZ glioma model, LDM TMZ significantly hampered tumor growth and angiogenesis and promoted apoptosis, compared to the conventional schedule. In the orthotopic U-87MG xenograft, TMZ even at a very low dose (0.25 mg/kg daily for 25 days) markedly reduced the microvessel density.	Kim et al. [40]
LDM TMZ (0.5 or 2 mg/kg/die p.o., 5 days per week for 21 days) vs. standard TMZ (30 mg/kg/die p.o. for 5 days, or 10 mg/kg/die, 5 days per week for 21 days)	TMZ-resistant RG2 glioma cells implanted s.c. in Fischer rats	TMZ significantly decreased the Treg/CD4+ T cell proportion when given at very low doses but not at high standard doses. Treg depletion alone, detected in LDM TMZ-treated rats, was not sufficient to significantly inhibit tumor progression, compared to control.	Banissi et al. [6]
LDM TMZ (1.77 and 0.9 mg/kg/day, for a total of 5 weeks) + weekly morphine	Orthotopic human U87MG-luc2 glioblastoma xenograft, implanted intracranially in Foxn1 nude mice	Addition of morphine enhanced the antitumor efficacy and the long-term response to the TMZ metronomic regimens.	Iorio et al. [43]
MEDIC (medium-dose intermittent chemotherapy) Cyclophosphamide (140 mg/kg i.p., every 6 day)	SCID mice bearing s.c. xenografts of rat 9L gliosarcoma, mouse GL261 glioma, and human U251 glioblastoma	The MEDIC schedule activated robust and persistent immune responses, as well as induced a striking and extended tumor regression. More effective than the corresponding daily low-dose metronomic regimen. Induction of a strong CD8+ T cell response leading to tumor disappearance and gain of immune memory in the GL261 glioma xenograft.	Chen et al. [44] Doloff et al. [45] Wu et al. [46,47]
Every 6-day metronomic cyclophosphamide (140 mg/kg) or temozolomide (140, 200, and 240 mg/kg)	Orthotopic GL261 glioma model in immunocompetent C57BL/6 mice	Longer survival rate in mice treated with both regimes than in control animals. In total, 140 mg/kg temozolomide was the best dosing in reduction of tumor volume and improving survival. No tumor eradication was achieved.	Ferrer-font et al. [48]
Metronomic TMZ (25 mg/kg × 10 days) vs. standard TMZ (50 mg/kg × 5 days) + immunotherapy	GL261 and KR158 intracranial glioma models in C57BL/6 mice	Addition of metronomic TMZ to PD-1 immune checkpoint inhibition preserved the survival benefit gained with anti-PD-1 therapy alone in the GL261 model, while it was abolished by standard temozolomide.	Karachi et al. [50]
Soft tissue sarcoma			
MTD doxorubicin (6 mg/kg once every 2 weeks), LDM doxorubicin (1.2 mg/kg, twice weekly for 4 weeks), DC101 (400 μg/dose every 3 days for a total of seven times), or combination	Female SCID mice (weight 18–22 g) harboring human RD rhabdomyosarcoma and SKLMS-1 leiomyosarcoma s.c. tumors	LDM doxorubicin alone was less effective than MTD doxorubicin. The combination of LDM doxorubicin and DC101 markedly reduced the volume of both SKLMS-1 and RD tumors, and the microvessel count, without overt toxicity.	Zhang et al. [53]
Bone sarcoma			
MTD methotrexate (1.35 g/kg i.v., 6 h infusion, once weekly, at week 0, 1, 5, and 6), adriamycin and cisplatin (10 mg/kg and 20 mg/kg, respectively; once weekly, at week 2 and 7), LDM methotrexate (1.2 mg/kg i.v., bolus, twice a week), combination of MTD and LDM schedules, for a total of 8 weeks	5-week-old SD rats bearing s.c. UMR 106 tumors	After the first 6 weeks of treatment, only the combination showed a protracted inhibition of tumor growth. Both MTD and combination schedules showed a much lower VEGF-A expression.	Zhu et al. [54]
Retinoblastoma			
In vitro: melphalan or topotecan in a conventional (0.001–1000 μM or 0.001–10.000 nM, respectively) single 72 h exposure vs. metronomic (0.0001–100 μM or 0.0001–1000 nM, respectively) 7-day continuous exposure In vivo: topotecan LDM topotecan (0.6 mg/kg i.p., 5 days a week) for 2 weeks) vs. MTD (3 mg/kg i.p., once a week) for 2 weeks	In vitro: commercial Y79 and WERI-RB1 and patient-derived HSJD-RBT-7 and HSJD-RBT-8 retinoblastoma cell lines. HUVEC and EPC human vascular endothelial cells In vivo: Y79 retinoblastoma s.c. xenografts in female athymic nude mice, weighing 18–22 g	Continuous administration increased the sensitivity to chemotherapy of retinoblastoma and endothelial cells. The heavily pretreated HSJD-RBT-8 cells had enhanced chemosensitivity to the metronomic schedule compared to the conventional one. Both treatment regimens led to cell death through apoptosis and/or necrosis in all cell lines. Metronomic schedules significantly inhibited in vitro tube formation in endothelial cells. LDM topotecan significantly lowered tumor volumes compared to the MTD protocol and control group, with a similar toxicity profile and no weight loss.	Winter et al. [57]
Acute lymphoblastic leukemia			
Dexamethasone (0.1, 1, and 7.6 μM) alone or in combination with low-dose arsenic trioxide (0.25 μM) for 72 h	GC-resistant ALL cell lines (CEM-C1-15, Jurkat, and MOLT-4), T-ALL, and precursor B-ALL cells from pediatric patients with poor response to prednisone	Low-dose arsenic trioxide significantly increased in vitro dexamethasone sensitivity. This combination reduced Akt phosphorylation, which is associated with an increase in Bad and decrease in XIAP protein.	Bornhauser et al. [60]
The 2-DG (0.2 to 10 mM) alone and in combination with dexamethasone (1 µM) for 24 h and 48 h	T-ALL cell lines: Molt-4 (GC resistance), Jurkat (GC resistance); CEM-C1-15 (GC resistance), CEM-C7-14(GC sensitive) B-ALL cell lines: Nalm-6 (GC sensitive), RS4:11 (GC sensitive) Burkitt lymphoma cell line: Raji (B-lineage, GC resistance)	Low-dose of 2-DG (1 mM) for 48 h induced apoptosis and cell-cycle arrest. Its combination with dexamethasone recovered the sensitivity of GC and produced a strong synergistic cytotoxic effect in GC-resistant Molt-4 and Raji cells.	Gu et al. [62]
L-ASNase 0.01 U/mL vs. 1 U/mL for 24 h	Cell lines: Jurkat and Reh (ALL), Jeko (mantle cell lymphoma), NK-YS (nasal-type NK-cell lymphoma)	Low-dose L-ASNase (0.01 U/mL) effectively killed Asn-dependent NK-YS cells, whereas clinically achievable intermediate doses (1 U/mL) induced robust apoptosis in Gln-dependent Jurkat and Jeko cell lines.	Sugimoto et al. [66]
Miscellaneous			
In vitro: increasing concentrations of evofosfamide with or without the presence of low-dose topotecan (20 nmol/L) In vivo: evofosfamide, (50 mg/kg daily i.p., 5 days/week), LDM topotecan (1 mg/kg daily by oral gavage, 5 days/week), and their combination	In total, 5 different neuroblastoma cell lines (CHLA-15, CHLA-20, CHLA-90, SK-N-BE(2), and SH-SY5Y) and 3 rhabdomyosarcoma cell lines (RH4, RH30, and RD) in vitro. Aggressive s.c. xenografts using two neuroblastoma cell lines [CHLA-20 and SK-N-BE(2)] and two rhabdomyosarcoma cell lines (RH4 and RD); metastatic (intravenous) SK-N-BE(2) neuroblastoma model in NOD/SCID mice	Low-dose topotecan enhanced the cytotoxic effect of evofosfamide in all cell lines in vitro. Combined treatment resulted in a better antitumor effect than both monotherapies and induced complete tumor regression after 2 weeks of treatment in each s.c. xenograft. It also improved survival in the SK-N-BE(2) metastatic model.	Zhang et al. [67]
In vitro: 96 h exposure of temozolomide (0.3–1000 μmol/L) with or without talazoparib (10 nmol/L) In vivo: temozolomide (30 mg/kg/day × 5 days) and talazoparib (0.25 mg/kg twice daily × 5 days) alone or in two different combinations: high-dose temozolomide (30 mg/kg/day × 5) + talazoparib (0.1 mg/kg twice daily × 5) or low-dose temozolomide (12 mg/kg/day × 5) + talazoparib (0.25 mg/kg twice daily × 5)	Pediatric Preclinical Testing Program (PPTP) cell line panel including a total of 23 cell lines of rhabdomyosarcoma, rhabdoid, Ewing sarcoma, neuroblastoma, glioblastoma, ALL, AML, ALCL, and NHL origin, for cytotoxic in vitro assays. In vivo pediatric xenografts: C.B-17 *scid*^−/−^ female mice for s.c. implantation of Wilms tumor, rhabdoid tumor, Ewing sarcoma, osteosarcoma, rhabdomyosarcoma, neuroblastoma, and non-glioblastoma brain tumors; BALB/c *nu/nu* mice for glioma models; female NOD.CB17-Prkdcscid/J mice for intravenous inoculation of human leukemia cells	In vitro, the combination resulted in a marked potentiation of temozolomide toxicity in Ewing sarcoma (50-fold) and ALL (30-fold) cell lines. In vivo, toxicity was comparable for both combinations. Both exhibited significant antitumor effects and induced total tumor regression in 5 of 10 Ewing xenografts, within 6 weeks of treatment. It was successful against xenografts with low MGMT expression (i.e., GBM2 glioblastoma and Rh28 rhabdomyosarcoma) and those with defective homologous recombination (i.e., KT-10 Wilms tumor).	Smith et al. [71]
In vitro: Topotecan for a total of 6 days, on three different ways of administration: continuous, for 8 h daily, or for 8 h every other day. In vivo: low-dose topotecan 0.6 mg/kg i.v. daily ×5 and 1.0 mg/kg every other day; increased dose 2 mg/kg daily ×5 and 3.3 mg/kg every other day (for 2 weeks, repeated every 21 days for 3 cycles)	In vitro: Daoy pediatric medulloblastoma and Rh30 pediatric rhabdomyosarcoma cells In vivo: Daoy and Rh30 s.c. xenografts in 4-week-old CBA/Caj tyhmectomized female mice	Topotecan IC_50_ values of ~2 nM for Daoy cells, while they increased with extending drug-free time in Rh30 cells. Rh30 xenografts regressed completely when treated with topotecan 0.6 mg/kg i.v. daily ×5. Increased doses of 2 mg/kg daily and 3.3 mg/kg every other day to obtain complete tumor regression of the less sensitive in vivo Daoy models.	Pawlik et al. [72]

2-DG, 2-deoxy-D-glucose; Akt, Protein kinase B; ALK, anaplastic lymphoma kinase; ALCL, Anaplastic large cell lymphoma; ALL, acute lymphoblastic leukemia; ALM, Acute myeloid leukemia; Bad, BCL2 associated agonist of cell death; Bcl-2, B-cell lymphoma 2; CAM, chorioallantoic-membrane; CEC, circulating endothelial cell; CEP, circulating endothelial progenitor; CM, conditioned medium; EC, endothelial cells; GC, glucocorticoid; GLUT3, Glucose transporter 3; HIF, hypoxia-inducible factor; IC50, half maximal inhibitory concentration; i.p., intraperitoneal; i.v., intravenous; KD, ketogenic diet; L-ASNase, L-asparaginase; LDM, low dose metronomic; mCTX, metronomic cyclophosphamide; MEDIC, medium-dose intermittent chemotherapy; MGMT, O-6-methylguanine-DNA methyltransferase; MTD, maximum tolerated dose; MYCN, Proto-Oncogene BHLH Transcription Factor; NB, neuroblastoma; NHL, Non-Hodgkin lymphoma; PD-1, programmed cell death protein 1; PEX, a fragment of human metalloproteases-2; p.o., per os; SASP, senescence-associated secretory phenotype; s.c., subcutaneous; TMZ, temozolomide; Treg; T regulatory cells; TSLC, tumor stem-like cells; VEGF, vascular endothelial growth factor; VEGFR-2, VEGF receptor 2; WT, wild type; XIAP, X-linked inhibitor of apoptosis protein.

## 4. Pharmacokinetic Studies on Metronomic Chemotherapy in Preclinical, Pediatric Tumor Models

The first preclinical pharmacokinetic (PK) study was performed by Zhou and colleagues [73] in SF188V+ glioma-bearing athymic rats receiving multiple i.v. administrations of temozolomide at a metronomic (3.23 mg/kg/day for 28 days) or standard (18 mg/kg/day for 5 days) dosing. The pharmacokinetic profile of temozolomide was linear, and dose- and time-independent. Indeed, the main PK parameters such as half-life (t_1/2_), volume of distribution (Vd), and systemic clearance of the drug were superimposable at the beginning and at the end of either metronomic or standard treatment. Moreover, a marked and similar decrease in tumor volume was observed in mice treated with both regimens, but the peak plasma concentration (C_max_) of metronomic temozolomide was considerably lower (7–8-fold) than that obtained after administration of the standard schedule, and it was maintained for a longer period of time [73].

Kumar and colleagues [29] have also investigated the pharmacokinetic interactions of metronomic topotecan in combination with pazopanib in a preclinical model of aggressive pediatric solid tumors. Pazopanib is a substrate of cytochrome P450 3A4 (CYP3A4) [74], whereas topotecan is an inhibitor of CYP3A4 [75]. However, no substantial difference was reported in the plasma concentration of topotecan or pazopanib among the group of animals treated with the drug combination and those treated with either agent alone. Therefore, interestingly, no relevant drug concentration alteration was detected between topotecan and pazopanib despite a theoretical metabolic drug interaction.

Interestingly, Chen and co-workers [44] carried out a pharmacokinetic analysis of plasma 4-OH-cyclophosphamide (active metabolite) levels in 9L glioma cell tumor-bearing mice administered with cyclophosphamide at 11.65 mg/kg p.o. daily, 70 mg/kg i.p. every 3 days, and 140 mg/kg i.p. every 6 days. The highest intermittent i.p. amount resulted in a C_max_ of 4-OH-cyclophosphamide almost 20-fold higher than that detected in mice treated with the lowest daily oral dose, although the total exposure was the same, and it was accountable for the activation of the strongest, broad-spectrum, antitumor innate immunity and tumor regression.

## 5. Clinical Studies on Metronomic Chemotherapy in Pediatric Patients

All the described clinical studies are summarized in Table 3.

### 5.1. Retrospective Studies

Roux and collaborators [76] conducted a monocentric retrospective study in 18 pediatric patients (7 males and 11 females) with pediatric low-grade glioma (pLGG). Induction treatment involved bevacizumab (10 mg/kg) alone or in combination with irinotecan (125 mg/m^2^) on days 1 and 15 of 2-week cycles for 6 months, or until the best clinical or radiological response was obtained; later, the patients received metronomic vinblastine (6 mg/m^2^ per week; in case of hematological toxicity, the dose could be reduced to 2 mg/m^2^ per week). Two patients had disease progression; therefore, it was not possible to include them in the analysis. Metastases were present in 2 out of 16 children, while 2 patients had undergone other treatments (1 line and 6 lines). Bevacizumab and irinotecan were given to 13 children, while bevacizumab alone was given to 3 children. After induction, 5 of 16 partial radiological responses and 11 of 16 stable diseases occurred. Induction lasted an average of 6.2 months, while maintenance lasted 12 months. Maintenance treatment had to be stopped in one patient due to grade 3 gastrointestinal toxicity with vomiting and abdominal pain. After 3.9 years (range 11 months–7.2 years) from the end of induction, 15/16 children were alive and 9/16 were progression-free. Disease progression occurred in 7 of 16 patients with a median time of 23 months: 3 children progressed during vinblastine maintenance, and 4 children after. The 2-year OS was 94%. Treatment tolerance was good overall. No patient experienced renal toxicity or hypertension during induction. Vinblastine was discontinued in one child after 3 months due to persistent grade 2 gastrointestinal toxicity (vomiting/nausea) during maintenance. Vinblastine dosage was decreased in 7 patients (n = 4, 4 mg/m^2^; n = 2, 3 mg/m^2^; n = 1, 2 mg/m^2^) due to hematological toxicities (grade 3 neutropenia). There were no peripheral neurotoxicity or long-term toxicities [76].

Seventy-four children with progressive, relapsed solid tumors, were treated by one of four different COMBAT chemotherapy regimens: COMBAT I (celecoxib, etoposide, temozolomide, isotretinoin) lasting 1 year; COMBAT II (celecoxib, etoposide, temozolomide, fenofibrate, cholecalciferol for the first year and celecoxib, cyclophosphamide, isotretinoin, fenofibrate, cholecalciferol for the second year) lasting 2 years; COMBAT IIS (celecoxib, vinorelbine, cyclophosphamide, cis-retinoic acid, fenofibrate, cholecalciferol) during 2 years for patients with soft tissue sarcomas and Ewing sarcomas; and COMBAT III (celecoxib, etoposide, temozolomide, fenofibrate, cholecalciferol, vitamin D3, bevacizumab for the first year and celecoxib, cyclophosphamide, isotretinoin, fenofibrate, cholecalciferol for the second year) lasting 2 years. Seventy-seven complete treatments were administered to 74 patients because 3 of them were re-challenged with a high-dose schedule. The investigators used these treatment schedules in a consecutive manner, displaying the progressive evolution of treatment protocols. The first 28 patients enrolled were treated with COMBAT I. Forty-four patients received COMBAT II and five patients COMBAT III. Overall, 43.1% of patients (median: 15.4 months) survived 2 years. The authors observed 62 patients with initially measurable diseases, 25 of whom progressed, but did not worsen during the follow-up period. The number of patients with measurable diseases who attained CR, PR, or SD after 6 months of COMBAT treatment had been defined as a clinical benefit, was 23%. Nine subjects showed a response 6 months after initiating COMBAT. During the follow-up, there were 50 (68%) deaths. In total, 24 patients survived: 6 (8%) patients had PD, 7 (9%) patients had PR/SD, and 11 (15%) had CR. Patients had a median time to response (CR/PR) of 6 months. Twelve children were affected by medulloblastoma (MBL)/primitive neuroectodermal tumors (PNET) and had the worst prognosis. They had a two-year OS of 33%; two children survived for a long time: 64 and 54 months. Twelve patients suffered from neuroblastoma, of which only two showed durable responses: one patient had a PR and progression-free survival (PFS) of 63 months, and one patient had a second very good PR at the last 12-month follow-up. Transient responses or stable disease with striking reduction in the dimension of the soft tissue mass have been shown by 8 patients. The patients with relapsed or progressive high-grade sarcoma did not have any long-term responses but had a palliative effect. Therapy led to response in 8 of 10 children with progressive LG tumors (LG glioma, giant bone cell tumors with lung metastasis, and LG sarcomas): tumor progression determined death, whereas a median PFS of 33 months was obtained in the remaining children. The treatment was administered on an outpatient basis and the tolerability was excellent. No therapy-related death or grade 3/4 non-hematological toxicity (except skin or hepatic toxicity) occurred. Grade 3 hepatic toxicities happened in only 8 children (11%). Grade 3 cheilitis occurred in 16 children (22%), when treated with cis-retinoic acid [77].

### 5.2. Case Series

Carcamo B. and Francia G. [78] reported a retrospective case series. Six children received metronomic treatment as a palliative cure because they had exhausted all therapeutic alternatives. Patient 1 was a male child diagnosed with metastatic rhabdoid tumor of the left kidney at about 3 years of age. At diagnosis, the metastases were located at the retroperitoneal level, in the lungs and bone marrow, as well as involving the renal vein and the inferior vena cava. The child, after courses of neoadjuvant chemotherapy and radiotherapy, had a progression of the disease in the lung and in bone marrow. At this point, the patient was treated with MC (etoposide 37.5 mg/m^2^/day orally on days 1–21, followed by cyclophosphamide 50 mg/m^2^/day orally on days 22–42, celecoxib 250 mg/m^2^ orally twice per day, and valproic acid 15 mg/kg/day divided into two doses): the rhabdoid tumor did not recur, and the pulmonary nodules remained stable. Subsequently, the child died from transplant-related infectious complications without showing, on imaging, signs of recurrent rhabdoid tumor, which therefore responded effectively for 3 years due to treatment. Patient 2 (male) had anaplastic ependymoma from the age of 6. The tumor was partially removed by surgery, and later treated with radiotherapy. After 6 months, there was a relapse in the brain and metastases to the leptomeninges and thoracic spine. Metronomic therapy was therefore initiated with oral etoposide (50 mg/m^2^/day for 21 days) alternating with oral CTX (50 mg/m^2^/day for 21 days), valproic acid, and continuous celecoxib (120 mg/m^2^ 2 times/day), subsequently modified with sulindac (8 mg/kg/day). The patient had a CR and no evidence of residual disease after 2 years of therapy; he received another 18 months of MC and was lost to follow-up after 8 years of diagnosis and 4 years of metronomic treatment without recurrence. Patient 3 (female) was diagnosed with medulloblastoma at the age of 10 months, and surgically treated in a definitive manner. Upon examination of the ventricular liquor, the presence of microscopic disease was detected; therefore, the girl was treated with chemotherapy and radiotherapy. Six months after the completion of the therapy, she had a microscopic recurrence in the cerebrospinal fluid. She was then given MC and after 5 months she achieved complete remission. After 5 years, the MC was discontinued. Patient 4 (female), age 5, had medulloblastoma localized to the fourth ventricle without desmoplastic or anaplastic features. She was treated with surgical resection, radiotherapy, and vincristine for adjuvant purposes. After 3 months, she had a first relapse, treated with temozolomide, irradiation, and surgical resection on the residual nodule. After another 3 months, a second relapse occurred. She was administered with MC (temozolomide alternating with CTX, celecoxib, and valproic acid), to which the patient partially responded. Ten months after the start of the metronomic treatment, an infiltrative brain tumor formed which led to the patient’s death 2 months later. Patient 5 (male) was diagnosed with metastatic neuroblastoma at age 9 and was treated with neoadjuvant chemotherapy, surgical resection, and finally with radiotherapy and isotretinoin. There was no evidence of residual disease at the end of treatment, but after 6 months there was a relapse treated with MC (etoposide alternating with CTX and continuous sulindac). Due to major hematological toxicities, it was necessary to reduce the dosage of etoposide and CTX, but the patient still showed a partial response at months 5 and 8. At the 11th month of treatment, there was a progression of the disease; thus, sulindac was replaced with celecoxib. After 2 months, the patient died. At 3 years of age, patient 6 (female) was diagnosed with type 2 neurocytoma of the spine. The tumor was treated surgically and later by irradiation, but a residual nodule remained. After 5 months, there was a progression of the disease with an increase in the size of the nodule and the formation of a second lesion; therefore, she received high-dose chemotherapy, developing important hematological toxicity and infectious complications, without obtaining any clinical benefit after 9 cycles. MC was then introduced (temozolomide alternating with CTX, valproic acid, celecoxib, and bevacizumab), and 3 months after the start of treatment there was a reduction in the size of the lesions. The metronomic treatment was gradually discontinued, but 10 months later it was necessary to resume it due to PD. It was possible to interrupt the treatment again, given that a stabilization of the disease was obtained; in the following 4 years of follow-up, the disease remained stable [78].

### 5.3. Phase I and Pilot Studies

Sterba and colleagues evaluated eight children with poor prognosis brain tumors treated by concomitant radiotherapy given 1 × 170 cGy, 5d/wk, for a total dose of 55/56 Gy, and temozolomide 90 mg/m^2^/day for 42 days. The primary toxicity, recorded around day 21, was myelosuppression. Vomiting was the most common non-hematologic toxicity, but these were rare, and no cases of dose-limiting toxicity occurred. Six patients responded to treatment. Two patients with high-risk medulloblastoma had the best responses. Furthermore, there were one more complete and three partial responses at the end of temozolomide treatment [79]. This study reported promising responses in children with medulloblastomas, which progress soon after the first surgery.

A prospective, pilot, single-center study conducted in Mali evaluated the efficacy and safety of a metronomic combination schedule based on vincristine, cyclophosphamide, and methotrexate in 12 children with refractory cancer (Wilms tumor and retinoblastoma were the most frequent diagnoses). There was no objective response, but a disease stabilization occurred in 7 patients (58%). They continued therapy for 15 to 24 weeks. In total, 6 subjects (50%) were still living after 39 weeks of median follow-up. For at least six months after the end of treatment, 3 patients (25%) showed stable disease. One patient developed grade 4 anemia, and one patient grade 4 non febrile neutropenia. No other grade 3 or 4 toxicities were detected [80].

An open label, nonrandomized, multi-center pilot study was designed to assess the safety of celecoxib (250 mg/m^2^ p.o. twice daily) combined with metronomic vinblastine (1 mg/m^2^ i.v. thrice weekly) or cyclophosphamide (30 mg/m^2^ p.o. daily) in patients under 21 years with a recurrent, refractory solid tumor. Patients with access were treated with vinblastine, and patients without access with cyclophosphamide. For the first week, the investigators administered only celecoxib to estimate safety and pharmacokinetics. In the second week, they added either vinblastine or cyclophosphamide. Thirty-three patients were enrolled, seventeen of whom were treated with vinblastine, and sixteen of whom with cyclophosphamide. Three patients of the vinblastine arm had a rapid disease progression, so were not evaluable and dropped out. The authors reported a small number of serious adverse events, none of which were likely associated with celecoxib, and observed no complete or partial responses. The median time to progression for the study was 8.5 weeks. Within 12 weeks, the progression of two-thirds of treated patients was observed, while 21% of patients received treatment for 12–24 weeks. In only 4 (13%) patients, a stable disease, lasting for 28 to 76 weeks, occurred. One patient presented a stable disease for over 15 months [81].

In a prospective, pilot, single-center study, 7 children from 3 to 21 years old with refractory cancer were administered with weekly vincristine (1.5 mg/m ^2^, days 1, 8, 15, and 22), daily cyclophosphamide (25 mg/m ^2^, days 1–21), twice-weekly methotrexate (15 mg/m^2^, days 21–42), and daily valproic acid (30 mg/kg) followed by one week rest. In the succeeding cycles, vincristine was given only on weeks 1 and 5. The authors obtained a mean duration treatment of 34 ± 31 weeks at the end of the observation period; two patients were alive with a mean follow-up of 9 ± 11 weeks. Two patients had partial responses, which lasted more than 2 years. One death after a quick progression occurred in a patient with metastatic osteosarcoma; he received treatment for more than a year. Two progressions and one loss of follow-up happened during the treatment. Regarding adverse events, there were 1 grade 4 anemia and 1 grade 4 non-febrile neutropenia [82].

Another study enrolled 20 patients under the age of 22 with recurrent or progressive poor prognosis tumors without other therapeutic options. The treatment scheme provided continuous oral thalidomide and celecoxib with an alternation of oral etoposide and cyclophosphamide every 21 days for 6 months at antiangiogenic doses. Rapid disease progression occurred in seven patients exposed to therapy for less than 3 months. One more patient had less than 3 months of therapy because of toxicity (i.e., thrombosis). Three patients continued treatment for 3–6 months before disease progression. Eight patients underwent the 6-month treatment, three of whom exhibited a radiographic partial response (1 glioma, 2 ependymomas). Seven out of eight patients decided to prolong the therapy beyond 6 months, supporting its good tolerability. Some transitory grade 1 and 2 toxicities were observed, but none of these led to discontinuation of treatment. Instead, only one patient with a grade 3 toxicity (i.e., deep venous thrombosis related to thalidomide) interrupted therapy. A longer-term toxicities assessment was performed through a growth and development evaluation. Only one of three menstruating female patients at study enrollment experienced amenorrhea. No growth retardation was identified. For example, a patient continued treatment for over 2 consecutive years and maintained a protracted partial response. At the beginning of treatment, he was 94.1 cm tall (50th percentile) and achieved a height of 106 cm (50th percentile) two years later [83]. These data support the low toxicity of the metronomic scheme.

Manji and colleagues [84] conducted a phase I clinical trial with the aim of establishing the maximum tolerated doses of metronomic topotecan and pazopanib, detecting the toxicities of the combined schedule, and describing the pharmacokinetics of oral metronomic topotecan and pazopanib prepared as a powder for oral suspension (PfOS) in 30 patients (age 2–21 years) with relapsed or refractory solid tumors. The initial doses of topotecan and pazopanib PfOS were 0.12 mg/m^2^ e 125 mg/m^2^, respectively. To increase the doses, the Rolling Six design was used to reach the doses of 0.4 mg/m^2^ and 160 mg/m^2^ of topotecan and pazopanib PfOS, respectively. At the end of the study, the recommended dose was topotecan 0.22 mg/m^2^/day and pazopanib PfOS 160 mg/m^2^/day. The median duration of treatment was 1.9 months (0.1–44.2). For the response, it was possible to evaluate 25 patients. Ten patients (4 neuroblastoma, 3 osteosarcoma, 2 Ewing sarcoma/PNET, and 1 medulloblastoma) had stable disease with a median duration of 6.4 months (1.7–45.1). The longest stable disease was 45 months, and the therapy was continued as compassionate use even after the study ended [84].

Ali and El-Sayed performed a prospective study, enrolling 64 subjects aged ≤18 with relapsed or refractory solid tumors. The patients were treated with at least 3 cycles of metronomic chemotherapy, each for 6 weeks plus a 1-week break (total 21 weeks), including celecoxib, cyclophosphamide, vinblastine, and methotrexate. In case of partial response or stable disease, the treatment was prolonged beyond 21 weeks. The patients with isolated local relapse or distant metastasis received radiotherapy in combination with metronomic chemotherapy. In total, 49 patients (77%) had a favorable response: 22 (34%) exhibited a partial response and 27 (42%) showed a stable disease. In contrast, 15 (23%) patients developed a progressive disease. Acute toxicities were mild: 26 (41%) patients experienced grade 1 hematologic toxicities, 10 (16%) non-hematologic toxicities, and 5 (8%) suffered a combination of toxicities. No toxicities were found in 23 patients (36%). The most common hematological toxicity was anemia (n = 16), and the most frequent non-hematological toxicity was neuropathy (n = 6). The 1-year overall survival (OS) rate was 62.3% after a median follow-up of 14 months. The 1-year OS was lower for patients who exhibited a progressive disease (17%) than for patients who achieved a stable disease (70%) or a partial response (82%) [85].

### 5.4. Phase II Studies

Thirteen subjects (ten male and three female) were included in a European multi-center, proof-of-concept, multi-arm phase I/II platform study in pediatric patients with relapsed/refractory cancer. The children were treated with nivolumab 3 mg/kg intravenously twice (day 1 and day 15) in each 28-day cycle, and with metronomic cyclophosphamide 25 mg/m^2^ orally twice daily, 1 week on and 1 week off. Patients were diagnosed from high-grade glioma (n = 3), neuroblastoma (n = 3), desmoplastic small round cell tumor-DSRCT (n = 3), alveolar rhabdomyosarcoma (n = 2), ependymoma (n = 1), and melanoma (n = 1). Metastases were present in 77% of patients (10/13). Previously, subjects were treated with a median of 3.5 treatment lines (range 1–5). During the study, 8 patients were irradiated at a dose of 20–40 Gy for locoregional metastases. There were 194 adverse events, but only 72 (37%) were probably drug-related: of these, 17 were grade 3 (15 of hematological type), whereas 5 were hematological grade 4. The 12 evaluable patients did not have a confirmed objective response. Stable disease occurred in 5 subjects. In the patient with DSRCT, there was a reduction in lesions observed at cycle 4 and confirmed at cycle 6, while in the patient with ependymoma the reduction in lesions observed at cycle 4 was not detectable at cycle 6. PD was the drop-out cause of all patients. The median PFS was 1.7 months (95% CI: 1.3–3.4), and the median OS was 3.4 months (95% CI: 2.2–13.5) [86].

In a multi-center, open-label, prospective two-stage phase II study, Robinson and colleagues evaluated an oral metronomic 5-drug regimen for children (age ≤ 21 years) with recurrent or progressive cancer. Cyclophosphamide and etoposide were administered in 21-day cycles and thalidomide, celecoxib, and fenofibrate were administered continuously. A total of 101 patients were enrolled in this study, and 97 began the treatment. Twenty-four patients (25%) appropriately performed 27 weeks of treatment without progressive disease (PD) or significant toxicity. Sixty-five (67%) interrupted therapy after PD, including three subjects who died of the disease. One death occurred because of complications of acute infection. Two subjects (2%) suspended therapy owing to toxicity. Five patients (5%) withdrew consent. Seven patients with ependymoma concluded therapy with stable disease (SD) or better. Twelve patients had low-grade glioma: three patients (25%) experienced PD within the first 9 weeks of the study, and nine patients (75%) experienced SD or better. One patient with an anaplastic glioneuronal tumor had an SD and proved an upcoming sustained complete response (CR). One subject with neurocytoma and chordoma had a PR. One patient with mixed malignant germ cell tumor, meningioma, hepatocellular carcinoma, and lymphangioma demonstrated an SD. Furthermore, one subject with choroid plexus carcinoma experienced PR. High-grade glioma and bone tumors had an unfavorable response rate. Three out of four patients affected by leukemia progressed soon after starting therapy. Six patients had medulloblastoma: three of them experienced the best response of SD or better, comprising one who showed a CR. One in three subjects with neuroblastoma ended therapy with SD. Most patients tolerated the treatment well. Hematological toxicities were the most common. One treatment-related death occurred for a patient with recurrent Ewing sarcoma who evolved Enterococcus faecalis bacteremia and neutropenia in week 4 of therapy. Two other patients dropped out of the study after the onset of toxicity: one with neutropenia and transaminase elevation, and one with a skin rash. The discontinuation of therapy led to the resolution of toxicities in both cases. Acute myelogenous leukemia (AML) was developed by an ependymoma patient exposed to the 5-drug regimen for 21 months. No other second malignancies have been reported [87].

A phase II clinical trial aimed to investigate the antitumor efficacy and the toxicity of metronomic weekly vinblastine (3 mg/m^2^, weeks 1–7), daily cyclophosphamide (30 mg/m^2^, days 1–21), twice weekly methotrexate (10 mg/m^2^, days 21–42), and twice daily celecoxib (100–200–400 mg, body weight <20 kg, 20–50 kg, >50 kg, respectively, days 1–56) in relapsed/refractory pediatric brain tumors. This schedule was followed by 13 days of rest from chemotherapy. The authors enrolled 29 patients: 8 patients with ependymoma in the first cohort; and 3 patients with medulloblastoma, 5 with high-grade glioma, 11 with low-grade glioma, and 2 patients with other tumors in the second cohort. An SD for 4 months was observed in one patient with progressive ependymoma. Instead, a tumor progression occurred in the other seven ependymoma patients after two cycles. In the second cohort, the authors enrolled 21 patients: 1 subject dopped out for early progression, and 1 for osteomyelitis. In the low-grade glioma (LGG) group, there were 2 partial responses, 6 stable diseases, and 2 progressive diseases. In the high-grade glioma (HGG) group, there were 1 stable disease, and 4 progressive diseases. In the group of other tumors, there were 4 progressive diseases. The median number of cycles was 6.8 (range 1–12). The authors deduced that this metronomic regimen was effective, especially in the LGG group. The hematologic toxicity, in particular neutropenia, was the most usual (n = 11 patients). Overall, 11 patients presented grade 3/4 neutropenia. Grade 4 febrile neutropenia occurred in two patients (1 ependymoma, 1 LGG). Five patients exhibited grade 3/4 lymphopenia. The investigators did not observe any grade 4 thrombocytopenia and non-hematological adverse events. The most common non-hematologic AE (n = 8 grade 2/3) was hepatic enzyme increase. Three patients presented grade 2/3 mucositis. Other non-hematological AEs included grade 2 rhinitis/pharyngitis, fatigue, keratitis/conjunctivitis, diarrhea, anal fissure, dizziness, paresthesia, and grade 3 hypophosphatemia and constipation. Five patients interrupted, temporarily, treatment for grade 3/4 toxicity (hepatic and/or hematological), leading to provisional lowering of the dose [88].

This clinical trial was the development of a previous study by André and collaborators, where 16 children (age 3–21) with refractory or relapsing tumors without other therapeutic options were treated with the above-described scheme of treatment. However, the protocol was mildly changed from the initial schedule because of toxicity and clinical outcome observed in the first 3 patients treated. In fact, when the tumor progressed, evidencing active neo-angiogenesis in the course of chemotherapy break, celecoxib administration was continued between two cycles. Moreover, the investigators added a seventh vinblastine dose on day 42. While patients were taking methotrexate, they developed mucositis; therefore, the dosage was reduced from 15 mg/m^2^ to 10 mg/m^2^. Overall, the last implemented version of the protocol was administered to 10 out of 16 patients. At the end of the observation period, 1 patient was still on treatment, and 7 patients were alive. A child with Hodgkin’s disease had the best response observed. The authors observed four stable diseases (25%), lasting 24 weeks or more, and four rapid tumor progressions (25%) in patients who had not completed the first cycle of treatment. Interestingly, they noted a rapid decline in pain and analgesic drug administration in 11 patients after the beginning of the metronomic therapy. Of greatest importance, tolerability was good. The toxicities were mostly hematological (83%). No child had alopecia and the investigators did not observe grade 3 or 4 nausea or vomiting. Toxicities did not lead to the treatment’s end. Three patients reduced vinblastine dosage by 30% because of one peripheral neurotoxicity case and two severe hematological toxicity cases. Two patients discontinued celecoxib, one because of renal insufficiency, and the other for hemoptysis related to lung metastasis. Grade 2 or 3 mucositis led to a reduction in the methotrexate dose in 4 patients [89].

Fifty patients from 4 to 25 years of age were enrolled in a prospective, multi-center, nonrandomized, noncomparative, open-label combination phase II study, but only 44 were evaluated [90]. Patients were divided into 4 groups based on the type of tumor they were suffering from: (i) neuroblastoma group (NBL) consisting of 18 subjects; (ii) soft-tissue sarcoma group (STS) composed of 7 patients; (iii) bone sarcoma group (BS) including 10 subjects; (iv) miscellaneous group (Misc.) involving 9 patients. The therapeutic scheme included 6-week cycles of MC and two treatment-free weeks for a total of 56 days. The drugs used were: twice daily celecoxib 100–200–400 mg (< 20 kg, 20–50 kg, > 50 kg, respectively) for 56 days, weekly intravenous vinblastine at a dose of 3 mg/m^2^ (days 1–8–15–22–29–36–43), and daily oral cyclophosphamide at a dose of 30 mg/m^2^/day during three weeks (days 1–21) alternating with oral methotrexate at a dose of 10 mg/m^2^ twice a week for three weeks (days 22–25–29–32–36–39–43). In 54% (24 out of 44) of patients, after 2 cycles of MC, no clinical signs of disease progression were detected, while in 46% (20 out of 44) of the subjects, it was necessary to interrupt the therapy due to a tangible PD. In total, 7 (4 NBL, 2 STS, 1 Misc.) out of 44 subjects (16%) clinically achieved a CR, PR, and SD from 2 cycles of treatment, whereas all BS patients (8 osteosarcoma and 2 Ewing sarcoma) had disease progression. The one-year PFS and the one-year OS of the whole cohort were 6.8% and 55.3%, respectively. In all, 33 out of 44 patients had 99 times grade 3/4 toxicity (24 events were grade 4). The most common toxicities were the hematological ones, in particular neutropenia: in fact, grade 3/4 neutropenia occurred 37 times in 24 subjects, while grade 3/4 thrombocytopenia occurred 6 times in 6 patients. Among the non-hematological toxicities, the most common was the elevation of transaminases: there was a grade 3 increase in liver enzymes in 6 subjects [90].

El Kababri and colleagues [91] performed a multi-center, prospective phase II study involving 98 pediatric patients with refractory/relapsing solid tumors or advanced disease. Patients were treated with 28-day cycles of oral cyclophosphamide (30 mg/m^2^) and oral etoposide (25 mg/m^2^) daily from day 1 to day 21, followed by 1 week off, and daily valproic acid (20 mg/kg) from day 1 to day 28. The subjects were suffering from neuroblastoma (n = 24), Ewing sarcoma (n = 18), osteosarcoma (n = 14), rhabdomyosarcoma (n = 14), and other miscellaneous diseases (n = 28). Metastases at diagnosis were present in 62 patients (63%). Patients received a median of 6 cycles (range 1–18) and, overall, tolerated the treatment well (no toxicity or maximum grade 1 toxicities in 95% of MC cycles). The only grade 3 or 4 toxicities observed were the hematological ones which occurred in 39 of 529 cycles. Toxicities did not necessitate a dose reduction. The 6-month OS was 40%; 1-year PFS was 19% and 1-year OS was 22%. Three children experienced a CR, 11 patients a PR, and 11 subjects had SD for more than 6 months. No relapses were observed 18 months after the end of treatment. Interestingly, 4 patients were relapse-free for 54 months. Ewing sarcoma, rhabdomyosarcoma, neuroblastoma, and Hodgkin lymphoma were most likely to respond to treatment, whereas osteosarcoma and Wilms’ tumor did not respond to treatment even in terms of disease stabilization. Quality of life, according to Karnofsky/Lansky scores, increased from 50% to 70% following MC [91].

### 5.5. Phase III Studies

In a multi-center, open label, randomized, controlled, phase III study, enrolling 371 patients aged 6 months to 21 years with rhabdomyosarcoma at high-risk of relapse, standard treatment (9 cycles of ifosfamide, vincristine, dactinomycin alone or in combination with doxorubicin, and surgery or radiotherapy or both) led to remission. After remission, patients were randomized 1:1 to discontinue treatment or proceed with maintenance chemotherapy (6 cycles of vinorelbine 25 mg/m^2^ i.v. on days 1, 8, and 15, and daily cyclophosphamide 25 mg/m^2^ p.o. on days 1–28, for 24 weeks), given on an outpatient basis. Overall, 5.75 months was the median time from randomization to the end of maintenance chemotherapy. Seven children interrupted treatment on parents’ request. At least one cycle modification occurred in 144 (80%) of 181 patients: the doses were lowered in the cases of neutropenia or thrombocytopenia in 74 (51%) patients, toxicity in 63 (44%), and other reasons in 7 patients (5%). Totally, 86.5% was 5-year overall survival in patients who received metronomic chemotherapy and 73.7% was 5-year overall survival in patients who discontinued treatment (HR 0.52 [95% CI 0.32–0.86]; *p* = 0.0097). Overall, 69.9% was 5-year disease-free survival in the group without further treatment, and 77.8% was 5-year disease-free survival in the group given maintenance chemotherapy. A relapse event occurred in 94 (25%) of 371 patients with similar distribution in the two groups. In particular, 6.9 months and 10.1 were the median times to relapse determined from the randomization date to the event in the arm given no further treatment, and in the arm with maintenance chemotherapy, respectively. There were 66 (18%) deaths: 42 (23%) of 186 in the subjects who stopped treatment, and 24 (13%) of 185 in the subjects still on chemotherapy. The patients died of relapse except for two patients in the group given no further treatment (one patient died of a surgical complication after a local relapse, and one was suicide) and two patients in the maintenance chemotherapy group (one patient with lung metastasis died of H1N1 infection, and one patient died of high-grade glioma, a second tumor appeared 69.7 months after rhabdomyosarcoma). The most common adverse event was grade 4 neutropenia, which occurred in 82 patients (45%). Fifty-six patients (31%) had grade 3 infection. Grade 3–4 leukopenia was reported in 136 (75%) of 181 patients, grade 3–4 neutropenia in 148 (82%) patients, anemia in 19 (10%) patients, and thrombocytopenia (1%) in 2 patients. One case (1%) of grade 4 non-hematological toxicity occurred. There were two treatment-related serious adverse events: one case of inappropriate antidiuretic hormone secretion, and one severe steppage gait with limb pain. The resolution of the events was reached, but in the patient with inappropriate antidiuretic hormone secretion, the treatment was permanently discontinued [92].

In a double-blinded, placebo-controlled, randomized clinical trial, 108 children aged from 5 to 15 years old were enrolled, affected by non-hematopoietic primarily extracranial solid tumors which progressed after at least 2 lines of chemotherapy. They were randomized 1:1 to receive placebo and best supportive care or metronomic chemotherapy and best supportive care. The eventual disease progression led to treatment stop. Metronomic chemotherapy consisted of two different alternating cycles; each cycle lasted 3 weeks. Cycle A was characterized by thalidomide, celecoxib, and etoposide administered daily; cycle B was characterized by thalidomide, celecoxib, and cyclophosphamide administered daily too. The placebo arm consisted of 52 patients; the metronomic chemotherapy arm consisted of 56 patients. At the cut-off date for data collection, the progression occurred in 107 patients (52 placebo; 55 metronomic chemotherapy), and death occurred in 107 patients (52 placebo; 55 metronomic chemotherapy). Overall, 2.9 months was the median follow-up for all the patients. Totally, 46 days and 49 days were the median PFS in the placebo group and in the metronomic chemotherapy group, respectively. In particular, 85 days was the OS in both groups. No complete responses occurred in either group. Eight patients had a stable disease and two patients had partial responses in the metronomic chemotherapy group. In a post hoc subgroup analysis, more than 3 cycles were administered to 40 patients and the PFS significantly increased (HR, 0.46; 95% CI, 0.23–0.93; *p* = 0.03). Furthermore, patients without a bone tumor benefited from metronomic chemotherapy for PFS (HR, 0.39; 95% CI, 0.18–0.81; *p* = 0.01) and OS (HR, 0.44; 95% CI, 0.21–0.90; *p* = 0.02). Grade 3–4 hematologic adverse events were the most common: anemia (7.1% vs. 11.7%), neutropenia (0% vs. 10.7%), thrombocytopenia (0% vs. 10.7%), and febrile neutropenia (0% vs. 8.8%) found in the placebo vs. metronomic chemotherapy group. The most common non-hematologic adverse event was mucositis (grade 1–2, 8.8%; grade 3–4, 5.3%) in the metronomic chemotherapy group. Eight patients (14.2%) needed a lowering of the dose, and nine patients had a delay in administration (metronomic chemotherapy group) [93]. The results obtained from the analysis of the subgroups showed an encouraging perspective in order to select the pediatric patients who can really have an advantage from the low-dose schedule.

In the same study, HRQoL (Health-Related Quality of Life) was also evaluated, using the PedsQOL Cancer module V.3, at baseline (A1), A2 (9 weeks or earlier if progressed), or A3 (18 weeks or earlier if progressed). No significant difference was observed in the change in the quality of life induced by each group from A1 to A2 in either mean total scores or individual domain scores. Moreover, no significant difference, defined as a 4.5-point improvement, was found either between bone sarcomas, other cancers, responders (≥9 weeks of treatment), or non-responders. This is in line with the survival outcomes observed in the study [94].

## 6. Biomarkers of Metronomic Chemotherapy in Pediatric Patients

Diagnostic, predictive, and prognostic biomarkers are urgently needed to select which pediatric patients could benefit most from metronomic therapy and to monitor treatment activity. These therapies are slow acting and may not induce tumor regression. Moreover, diverse biomarkers may be needed according to the treatment and disease type.

Conflicting results have been published on cytokines as biomarkers of metronomic in the pediatric setting, some in favor and others opposing their role in predicting the effectiveness of metronomic therapies [81,83,87,95]. Driven biomarkers, as cytokines, may not be the best parameters, as there are different upstream and downstream pathways involved, and a single parameter (i.e., VEGF) may not be representative of all.

First, the previously described study of Kieran and colleagues [83] also evaluated serum, plasma, and urine levels of VEGF; basic fibroblast growth factor (bFGF); thrombospondin-1 (THBS1); and endostatin. Baseline THBS1 levels in serum above 75 μg/mL were associated with extended disease-free survival (>1 year) in three patients, while VEGF, bFGF, and endostatin levels during therapy did not show a change relative to baseline values. On the contrary, four subjects with baseline levels below 75 μg/mL demonstrated early progression. Nevertheless, THBS1 levels did not increase further during therapy, independently of radiographic response, stable disease, or rapid tumor progression [83].

Similarly, Stempak and collaborators assessed several circulating plasma proteins (i.e., VEGF, bFGF, THBS1, endostatin, soluble vascular and intercellular cell adhesion molecule) as surrogate markers of angiogenic activity in children with recurrent, refractory solid tumors continuously exposed to celecoxib and either low-dose vinblastine or cyclophosphamide. However, these markers were highly variable and showed no significant correlation with disease progression or stable disease [81].

André and colleagues monitored the influence of the maintenance of metronomic-like chemotherapy on angiogenic cytokines (i.e., VEGF, VEGFR-1, Ang-2), THBS1, Treg, and endothelial biomarkers (i.e., CEC, EPC, EMP), in 47 children with ALL. Along with a remarkable increase in THBS1 levels, a statistically significant reduction in EPC and EMP counts was detected during this stage. No appreciable variation was observed in other angiogenic markers or in the Treg fraction [95].

Furthermore, biomarkers of angiogenesis were evaluated in a pediatric population treated with sirolimus in combination with metronomic oral topotecan and cyclophosphamide. A statistically significant reduction has been observed in THBS1 and soluble VEGFR-2 plasma levels at 21 days if compared to baseline, demonstrating that this regimen can regulate angiogenic pathways [96].

Robinson and co-workers [87] conducted a surrogate marker analysis evaluating VEGF, bFGF, endostatin, and THBS1 levels in blood and urine samples at baseline and every ninth week, in children with recurrent or progressive cancer administered with the aforementioned 5-drug oral regimen. Interestingly, patients who completed therapy showed significantly higher baseline serum THBS1 levels than patients who discontinued treatment (median 9163 vs. 4299 ng/mL), suggesting an association with tumor susceptibility to antiangiogenic therapy. No statistical differences between completer and non-completer patients have been reported concerning the other biomarkers analyzed (VEGF, bFGF, and endostatin) [87].

Recently, Pramanik and colleagues evaluated serum VEGF and THBS1 concentrations in 108 pediatric patients with progressive malignancies who randomly received metronomic chemotherapy or placebo [97]. They found no differences for both these biomarkers at baseline and two other time points (week-9 (A2) and week-18 (A3) or earlier if progressed) between the two arms, as well as at the change from baseline to A2 in each group. Responders to metronomic chemotherapy, who completed at least 3 cycles, displayed significantly inferior levels of mean baseline VEGF [659.7 vs. 1143.9 μg/mL] and a substantial decrease in THBS1 from baseline to A2 [−4.43 vs. 1.7 μg/mL], as opposed to non-responders. Responders in the placebo group did not show such alterations [97].

Based on these conflicting results, VEGF and THBS1 cannot yet be considered valid biomarkers of the response to metronomic chemotherapy in pediatric patients.

Moreover, studies have investigated the role of other potential biomarkers such as HIF-1α expression [98], and factors implied in resistance to metronomic chemotherapy (i.e., cathepsin B, ANXA3) [99].

In particular, Tho and collaborators demonstrated that among the clinically used topoisomerase II (TOP2)–targeting drugs evaluated in their study (i.e., mitoxantrone, doxorubicin, and etoposide), only mitoxantrone induced a potent, dose- and time-dependent, inhibition of HIF-1α expression under hypoxic conditions, similar to that caused by cycloheximide. In vitro experiments using HIF-1α mRNA corroborated that mitoxantrone inhibited the HIF-1α translation step. Noteworthy, mitoxantrone treatment was associated with increased polysome-bound HIF-1α and decreased VEGF-A mRNA levels [98].

Immune cells such as Treg cells and myeloid-derived suppressor cells may also be considered as potential biomarkers.

However, the studies mentioned above have several limitations. First, the small groups of proteins evaluated in these studies do not represent the total effect of all the regulators of angiogenesis. Furthermore, the absence of standard reference ranges to detect circulating proteins could lead to a problematic interpretation of the results.

It is also difficult to identify potential biomarkers for metronomic chemotherapy because of the various mechanisms of action, multiple drug combinations, and many clinical settings used. However, the search for new potential biomarkers may be plausible in the future thanks to the broad accessibility of next-generation sequencing. Further biomarkers could be considered for measuring the efficacy of metronomic chemotherapy such as circulating cell-free DNA, circulating endothelial cells, and circulating endothelial precursor cells and micro-particles. Future studies including homogenous patient populations and focusing on validating surrogate markers to monitor treatment activity are needed.

## 7. Conclusions and Future Perspective

The costs of cancer treatment have increased worldwide over the years, especially with the advent of immunotherapy and targeted therapy. Within the USA, the overall national costs of cancer care are predicted to rise by over 30% from 2015 to 2030, corresponding to over $245 billion, based on population growth [100].

**Figure 2:**
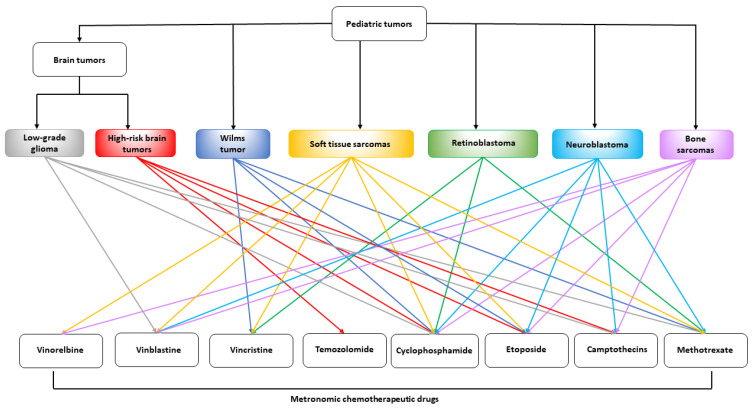
Chemotherapeutic drugs metronomically administered in pediatric clinical trials in different tumor types.

Metronomic chemotherapy could represent a valid method to reduce the economic burden of anticancer therapy in the pediatric setting. In fact, most metronomic regimens involve the use of inexpensive, old drugs available for oral administration (Figure 2), thus reducing costs for hospitalization, intravenous injections, risk of infection, and travel or accommodation outlay for patients and their families who must visit well-equipped health centers in large and distant cities. Side effects associated with low-dose metronomic chemotherapy are usually limited compared with MTD therapy. Therefore, treatment complications and the frequency of monitoring tests could be reduced, resulting in improved quality of life, both for patients and caregivers, and further lowering of costs for cancer treatment without impairing clinical outcomes [10].

Maintenance therapy in pediatric patients is administered to hamper cancer progression and/or relapse after successful initial therapy. It aims at extending survival while preserving the quality of life [101]. Metronomic chemotherapy represents a good option for maintenance therapy because it is usually well-tolerated, and it can be given orally and at a relatively low cost. Moreover, its activity on angiogenesis, cancer stem cells, and the regulation of the immune system allows the possibility to overcome resistance and delay tumor progression [99].

However, long-term morbidities such as the development of secondary tumors have become a relevant issue for pediatric patients. Chemotherapy use including alkylating agents has been correlated with the increased risk of hematologic malignancies [102]. Therefore, it will be of interest to deeply evaluate the impact of the total dose of a metronomic cyclophosphamide-based schedule, which may expose pediatric individuals to the risk of developing secondary tumors. Moreover, intensive surveillance and counselling of these patients are necessary to monitor and anticipate secondary tumor diagnosis and treatment [102].

All metronomic phase I and II studies published in the last decade reveal good tolerance and acceptable side effect profiles in outpatient care without affecting the normal growth of children [83]. Most of the evidence comes from preclinical studies or phase I and phase II clinical studies performed in the relapse/refractory setting. Unfortunately, the lack of large, randomized phase III trials and of reliable biomarkers made the clinicians question using a low-dose, continuous regimen as the standard of care in pediatric oncology. Therefore, further phase III studies are urgently needed to validate the role of metronomic chemotherapy in the present and future disease control [9]. As metronomic chemotherapy is assumed to display its effect also by immunomodulation, we might expect interesting results from a combination of immunotherapies. Furthermore, a proper combination with radiotherapy, MTD chemotherapy, and novel targeted drugs should be tested in future research [9].

The most plausible approach may be to proceed with investigations and gradually integrate metronomic treatments into the current clinical practice, taking advantage of their low toxicity, oral administration and, therefore, the feasibility of a more comfortable, home-based treatment.

## Figures and Tables

**Table 1:** Main and secondary keywords used for the literary search.

Main Key Words	Secondary ^a^ Key Words
Metronomic	Preclinical study
Low dose	Cell culture
Chemotherapy	Animal model
Pediatric	Clinical study
Childhood	Randomized controlled trial
Cancer	Observational study
Tumor	Efficacy
Neoplasia	Toxicity
	Angiogenesis
	Biomarkers

^a^ Secondary key words were utilized in combination (by using “AND”) with the main key words. Main keywords were combined by using “OR”, reported in left column.

**Table 3:** Clinical studies on metronomic chemotherapy in pediatric patients.

Disease	N° of Patients	Type of Study	Metronomic Regimen	Main Results	Reference
Low-grade glioma (pLGG)	18	Retrospective	Bevacizumab with or without irinotecan + vinblastine/vinorelbine	2-year OS: 94%	Roux et al. [76]
Progressive or relapsed solid tumors	74	Retrospective	COMBAT I, COMBAT II, COMBAT IIS, COMBAT III	43.1% (median: 15.4 months) was 2-year OS	Zapletalova et al. [77]
Rhabdoid tumor	6	Case series	Etoposide + cyclophosphamide + celecoxib + valproic acid	SD (death from bone marrow transplant-related infectious complications)	Carcamo B. and Francia G. [78]
Anaplastic ependymoma			Etoposide + cyclophosphamide + celecoxib + valproic acid	CR	
Medulloblastoma			Etoposide + valproic acid	CR	
Medulloblastoma			Temozolomide + cyclophosphamide + celecoxib + valproic acid	PD	
Neuroblastoma			Etoposide + cyclophosphamide + sulindac/celecoxib	Relapse	
Cervicomedullary tumor			Temozolomide + cyclophosphamide + valproic acid + celecoxib + bevacizumab	SD	
Poor prognosis brain tumors	8	Pilot	Temozolomide 90 mg/m^2^/day for 42 days	Six patients responded to treatment	Sterba et al. [79]
Refractory solid tumors	12	Pilot, prospective	Vincristine, cyclophosphamide, and methotrexate	Disease stabilization occurred in 7 patients (58%)	Fousseyni et al. [80]
Recurrent or progressive solid tumor	33	Pilot	Celecoxib + vinorelbine or cyclophosphamide	Median time to progression was 8.5 weeks (range: 3 to 62.5 week) Four (13%) patients had a stable disease with durations of 28 to 76 weeks	Stempak et al. [81]
Refractory cancer	7	Pilot, prospective	Vincristine, cyclophosphamide, methotrexate	Mean duration treatment: 34 ± 31 weeks PR: 2 patients	Traore et al. [82]
Recurrent or progressive poor prognosis solid tumors	20	Feasibility trial	Four-drug regimen: thalidomide + celecoxib + alternating cyclophosphamide/VP16 every 21 days for 6 months	Eight patients completed the six-month therapy	Kieran et al. [83]
Relapsed or refractory solid tumors	30	Phase I	Topotecan + pazopanib	The recommended dose was topotecan 0.22 mg/m^2^/day and pazopanib PfOS 160 mg/m2/day In total, 10 patients (4 neuroblastoma, 3 osteosarcoma, 2 Ewing sarcoma/PNET, and 1 medulloblastoma) had stable disease with median duration of 6.4 months (1.7–45.1) The longest stable disease was 45 months	Manji et al. [84]
Relapsed or refractory solid tumors	64	Prospective	Celecoxib, cyclophosphamide, vinblastine, and methotrexate	Forty-nine patients (77%) had a favorable response (PR and SD) 1-year OS: 62.3%	Ali A.M. and El-Sayed M.I. [85]
High-grade glioma, neuroblastoma, desmoplastic small round cell tumor-DSRCT, alveolar rhabdomyosarcoma, ependymoma, melanoma	13	Phase II	Nivolumab + cyclophosphamide +/- radiotherapy at discretion of physician	None of patients had confirmed objective response Stable disease occurred in 5 subjects Median PFS = 1.7 months (95% CI: 1.3–3.4) Median OS = 3.4 months (95% CI: 2.2–13.5)	Pasqualini et al. [86]
Recurrent or progressive tumors	101	Phase II, prospective	Five drugs: cyclophosphamide, etoposide, thalidomide, celecoxib, and fenofibrate	Twenty-four patients (25%) completed 27 weeks therapy	Robison et al. [87]
Relapsed/refractory pediatric brain tumors	29	Phase II	Four drugs: celecoxib, vinblastine, cyclophosphamide, and methotrexate	Median number of cycles was 6.8 (range 1–12) Good response in LGG patients	Verschuur et al. [88]
Refractory or relapsing tumors	16	Pilot	Vinblastine, cyclophosphamide, methotrexate, and celecoxib	Disease stabilizations (25%) that lasted 24 weeks or more	André at al. [89]
Neuroblastoma, soft-tissue sarcoma, bone sarcoma, miscellaneous	50	Phase II, prospective	Vinblastine, cyclophosphamide/methotrexate, celecoxib	1-year PFS = 6.8% 1-year OS = 55.3%	Heng-Maillard et al. [90]
Refractory/relapsing solid tumors, or advanced disease	98	Phase II, prospective	Cyclophosphamide + etoposide + valproic acid	6-month OS = 40% (95% CI) 1-year OS = 22% 1-year PFS = 19%	El Kababri et al. [91]
Rhabdomyosarcoma at high-risk of relapse	371	Phase III	G1: vinorelbine and cyclophosphamide G2: placebo	In G1 group: 86.5% (95% CI 80.2–90.9) was 5-year overall survival 77.8% (70.8–83.4) was 5-year disease-free survival	Bisogno et al. [92]
Non-hematopoietic primarily extracranial solid tumor progressive after treatment with at least 2 lines of chemotherapy	108 G1: 56 patients G2: 52 patients	Phase III	G1: thalidomide, celecoxib, and etoposide/cyclophosphamide G2: placebo	In the G1 group: 49 days (95% CI, 43–59 days) was the median PFS 85 days was the OS SD = 8 patients PR = 2 patients Good response in patients without a bone tumor	Pramanik et al. [93]

COMBAT I (celecoxib, etoposide, temozolomide, isotretinoin); COMBAT II (celecoxib, etoposide, temozolomide, fenofibrate, cholecalciferol for the first year and celecoxib, cyclophosphamide, isotretinoin, fenofibrate, cholecalciferol for the second year); COMBAT IIS (celecoxib, vinorelbine, cyclophosphamide, cis-retinoic acid, fenofibrate, cholecalciferol); COMBAT III (celecoxib, etoposide, temozolomide, fenofibrate, cholecalciferol, vitamin D3, bevacizumab for the first year and celecoxib, cyclophosphamide, isotretinoin, fenofibrate, cholecalciferol for the second year); CR, complete response; DSRCT, desmoplastic small round cell tumor; G1, group1; G2, group 2; OS, overall survival; PfOS, powder for oral suspension; PFS, progression-free survival; PD, progressive disease; pLGG, pediatric low-grade glioma; PNET, Primitive Neuro-Ectodermal Tumors; PR, partial response; SD, stable disease; VP16, etoposide.
